# Autophagy Is Required for Strawberry Fruit Ripening

**DOI:** 10.3389/fpls.2021.688481

**Published:** 2021-08-27

**Authors:** José F. Sánchez-Sevilla, Miguel A. Botella, Victoriano Valpuesta, Victoria Sanchez-Vera

**Affiliations:** ^1^Unidad Asociada al CSIC de I+D+i Biotecnología y Mejora en Fresa, Instituto Andaluz de Investigación y Formación Agraria y Pesquera (IFAPA), Centro IFAPA Málaga, Junta de Andalucía, Málaga, Spain; ^2^Departamento de Biología Molecular y Bioquímica, Instituto de Hortofruticultura Subtropical y Mediterránea (IHSM), Universidad de Málaga-Consejo Superior de Investigaciones Científicas, Málaga, Spain

**Keywords:** autophagy, fruit ripening, strawberry, plant development, senescence, vascular tissue

## Abstract

Autophagy is a catabolic and recycling pathway that maintains cellular homeostasis under normal growth and stress conditions. Two major types of autophagy, microautophagy and macroautophagy, have been described in plants. During macroautophagy, cellular content is engulfed by a double-membrane vesicle called autophagosome. This vesicle fuses its outer membrane with the tonoplast and releases the content into the vacuole for degradation. During certain developmental processes, autophagy is enhanced by induction of several autophagy-related genes (*ATG* genes). Autophagy in crop development has been studied in relation to leaf senescence, seed and reproductive development, and vascular formation. However, its role in fruit ripening has only been partially addressed. Strawberry is an important berry crop, representative of non-climacteric fruit. We have analyzed the occurrence of autophagy in developing and ripening fruits of the cultivated strawberry. Our data show that most *ATG* genes are conserved in the genome of the cultivated strawberry *Fragaria x ananassa* and they are differentially expressed along the ripening of the fruit receptacle. ATG8-lipidation analysis proves the presence of two autophagic waves during ripening. In addition, we have confirmed the presence of autophagy at the cellular level by the identification of autophagy-related structures at different stages of the strawberry ripening. Finally, we show that blocking autophagy either biochemically or genetically dramatically affects strawberry growth and ripening. Our data support that autophagy is an active and essential process with different implications during strawberry fruit ripening.

## Introduction

Autophagy is a general mechanism in eukaryotes that maintains the cell homeostasis through the degradation of intracellular targeted organelles, proteins, and major compounds (Marshall and Vierstra, 2018). Several autophagic routes have been described in animals, fungi, and plants, with the best known initially referred to as macroautophagy (hereafter named as autophagy) (Galluzzi et al., 2017). In this pathway, a double membrane structure is initiated that grows until its complete closure, engulfing cytoplasmic material and giving rise to a structure called autophagosome. The autophagosome fuses its outer membrane with the tonoplast, releasing its content into the vacuole where it gets degraded by vacuolar proteases and hydrolases (Wen and Klionsky, 2016). Under favorable conditions, low levels of autophagy flux serve as a housekeeping function by clearing obsolete cytoplasmic content, whereas, during periods of stress or starvation, autophagy flux is enhanced to promote cell survival by recycling damaged proteins and organelles and thereby reallocating energy and building blocks for biosynthetic processes (Rabinowitz and White, 2010). Autophagy is driven by a set of core autophagy-related proteins (ATG) first described in yeast and conserved in almost all eukaryotes (Tsukada and Ohsumi, 1993; Meijer et al., 2007). They participate in all the stages of the pathway, from cargo recognition and autophagosome formation to docking to the vacuole and release of the content (Marshall and Vierstra, 2018).

In plants, it has been shown that autophagy plays a crucial role not only in resistance to stress but also in development, thanks mostly to studies performed in *Arabidopsis* (Su et al., 2020). Lines lacking functional ATG proteins show an overall reduction in plant fitness, including reduced growth and fecundity, accelerated senescence, as well as high susceptibility to biotic and abiotic stresses (Li and Vierstra, 2012). Autophagy has been associated with the metabolic adjustment taking place during the plant developmental transitions (Michaeli et al., 2016). The maintenance of adequate supplies of nutrients needed for all developmental processes requires the release of nutrients from stores and the recycling of macromolecules, in addition to nutrition acquisition and synthesis. Autophagy is one recycling route that might provide essential nutrients for its reuse by the plant cell (Marshall and Vierstra, 2018).

In the last years, given the relevance of autophagy for plant fitness, the study of this catabolic process in crops has gathered more interest. To date, autophagy in crops has been studied in the context of several developmental processes, such as leaf senescence, seed and reproductive development, and vascular formation (Tang and Bassham, 2018). A highly relevant developmental process that is absent in *Arabidopsis* but is extremely important for agriculture is the ripening of fleshy fruits. Ripening of fleshy fruits comprises changes in color, size, hardness, and production of metabolites that generate fruity smells and sweetness (Giovannoni, 2001). Although, a plethora of molecular players have been described to be related to this developmental process using tomato as model (Osorio et al., 2013), very little is known about autophagy in the context of fruit ripening. Transcriptomic analyses on grape berry skin revealed that a set of *ATG* genes (*ATG18g, ATG9, ATG11*, and *ATG2*) showed a higher expression concomitant with ripening, supporting a role for autophagy in fruit senescence (Ghan et al., 2017). A recent study, analyzing autophagy related genes and proteins (*ATG8, ATG4, ATG5, ATG9*, and *NBR1*), and confirming the presence of autophagic-like structures, suggested that autophagy occurs during ripening in pepper (López-Vidal et al., 2020). However, the importance of autophagy during fruit ripening has not been elucidated yet.

Strawberry is a non-climacteric achenetum-type of fruit that develops into a fleshy receptacle, in which the dry achenes are embedded (Liu et al., 2020). The receptacle of the ripe fruits is a major contributor to the mass of the fruit and the main determinant of the quality parameters. During the development of the fruit receptacle the cellular changes are well-defined, from cell division to cell expansion (Havis, 1943), as well as the metabolic changes (Fait et al., 2008) and gene expression (Sánchez-Sevilla et al., 2017). In the present work, we have studied whether autophagy could play a role in the strawberry fruit ripening process. We have analyzed the changes in expression of autophagy-related genes during ripening of the cultivated strawberry *Fragaria x ananassa*, as well as the ATG8 protein content and its lipidation, as a required step for the formation of the autophagosomes. Here, we report that most of the *ATG genes*, as well as the regulatory *ATAF1* and the autophagy marker *NBR1*, show a significant level of expression in the receptacle, and this expression changes along the ripening process. The amount of lipidated ATG8 peaked at the white and ripe stages of development, supporting the existence of two autophagic waves along strawberry ripening. At the cellular level, we have identified the presence of autophagy-related structures at different stages of the receptacle ripening, including autophagosomes. Finally, we show that blocking autophagy in fruits, either biochemically or genetically, dramatically affects the ripening process. Taking all this data together, we propose that autophagy is a key process in strawberry ripening with clear implications in vascular development and senescence of the fruit.

## Materials and Methods

### Plant Material and Growth Conditions

Plants of the short-day variety *Fragaria x ananassa* Duch (cv. “Camarosa”) and the neutral day *Fragaria x ananassa* cv. “San Andreas” were grown under greenhouse conditions (IHSM, Málaga, Spain). In the case of *F x ananassa* cv “San Andreas” a supplement of light was applied from 8:00 to 20:00 and from October to February when sunlight intensity was below 5,000 lux.

For developmental assays, fruits were harvested at six different developmental stages corresponding to green (G), white (W), turning (T), red (R), ripe (Rp), and over-ripe (Or). We determined each stage based on the following hallmarks: (i) green: small green receptacle with green achenes, (ii) white: white receptacle with yellow achenes, (iii) turning: semi-red receptacle, (iv) red: red receptacle, (v) ripe: hard and dark red receptacle, and (vi) over-ripe: soft and dark red receptacle. Examples of the stages can be found in **Figure 5A** and Supplementary Figure 2A. Each sample corresponded to a pool of 4–5 fruits that were harvested and flash-frozen in liquid nitrogen. Achenes and receptacle develop coordinately but suffer different developmental programs as can be deduced by their different appearance and metabolic composition (Fait et al., 2008). In order to be sure that our molecular analysis corresponded to receptacle exclusively, we removed achenes from frozen fruits with the help of a scalpel.

### Identification of Strawberry Autophagy-Related Genes, Read Mapping, Expression Analysis, and Sequence Alignments

In order to identify the orthologs of autophagy-related genes in *F. x ananassa*, first a BLAST of the *Arabidopsis ATG* genes on the well-annotated genome of the diploid *Fragaria vesca* was performed (https://www.rosaceae.org/species/fragaria/fragaria_vesca). Retrieved sequences were confirmed by analyzing their protein domains with pHMMER and comparing it with the Arabidopsis orthologs (https://www.ebi.ac.uk/Tools/hmmer/search/phmmer). The e-value cutoff used in the gene search was 0.001, however the genes confirmed with HMMR as putative orthologs showed an e-value below 1e-40 in all cases.

Correspondences between *Arabidopsis, F. vesca* (v4.0.a1) (Edger et al., 2018), and *F. ananassa* (v1.0.a2) *ATG* genes were established with BLAST.

Next, we investigated the expression of these genes using the transcriptome data previously generated by our group that include fruits of *F. x ananassa cv*. “Camarosa” at different ripening stages (green, white, turning, and red) (Sánchez-Sevilla et al., 2017). Reads mapping and differential gene expression analysis were performed according to the Tuxedo protocol workflow for RNA-seq differential expression analysis (Hisat2/Cufflinks/CummRbund) using the default parameters (Trapnell et al., 2012). As reference genome we used the *Fragaria x ananassa “*Camarosa” Genome Assembly v1.0 (Edger et al., 2019) and as reference annotation *Fragaria x ananassa “Camarosa” Genome v1.0.a2 (Re-annotation of v1.0.a1)* (Liu et al., 2021) available at GDR (https://www.rosaceae.org/species/fragaria/fragaria_x_ananassa). Statistical analysis of RNAseq data was performed with the false discovery rate (FDR) method after Benjamini-Hochberg correction for multiple-testing. For *ATG8*, 36 gene sequences were retrieved after BLAST against *F x ananassa* genome, however due to poor assembly of the genomic sequence in the case of one of them (FxaC_27g11741) we decided not to include it in the subsequent analysis.

All bioinformatics processes were developed at the Supercomputing and Bioinnovation Center of the University of Malaga (https://www.scbi.uma.es/site/). Reads are stored at the European Nucleotide Archive (https://www.ebi.ac.uk/ena) with the study reference PRJEB12420. Nucleotide and amino acid sequences were aligned, clustered, and analyzed with MAFFT online service (Katoh et al., 2018). Alignment data can be found in Supplementary Material (Data Sheet 1). The phylogenetic tree was inferred by the Neighbor-Joining method with a bootstrap analysis. A total of 1,000 replicates were used to estimate the reliability of internal nodes. The evolutionary distances were computed using the Maximum Composite Likelihood method. Evolutionary analyses were conducted in MEGA X (Stecher et al., 2020) and tree was displayed using FigTree (http://tree.bio.ed.ac.uk/software/figtree/).

### Transmission Electron Microscopy

*F x ananassa* cv. “Camarosa” fruits were cross sectioned and 2 mm thick pie sections containing the epidermis, cortex, and pith were dissected with a scalpel. Samples were fixed in 2,5% glutaraldehyde (Acros organics, Thermo Fisher Scientific) and 50 mM sodium cacodylate buffer (pH 7,4) under vacuum for 2 × 15 min at 4°C and left in fixative o/n. Fixed samples were washed with buffer three times for 10 min and then post- fixed in buffered 1% OsO4 (Electron Microscopy Sciences, 19150) for 60 min. After washing them in water, samples were incubated with 2% uranyl acetate (aqueous) (Electron Microscopy Sciences, 22400) for 2 h and then washed with water. After samples were dehydrated through an ethanol series (Panreac, 141086.1214; 50, 70, and 95% each step 30 min; 100% for 30 min and 100% for 30 min) and incubated in a solution of London Resin White (Electron Microscopy Sciences, 14381-UC) and ethanol (1:1) overnight. Samples were incubated in pure London Resin White for 5 h and London Resin White overnight; polymerization was performed at 65°C for 24 h. Ultrathin sections (50–70 nm) were obtained with an ultramicrotome Leica EM UC7/FC7. Sections were examined with a JEM-1400 (Jeol, Málaga, Spain) transmission electron microscopy.

### Western Blotting

Protein extractions were performed from de-achenized receptacles grounded in liquid nitrogen. In all cases, 600μl of extraction buffer [Tris-HCl 50 mM pH 7,5, Nonidet N-40, P9599 protease inhibitors (Sigma) and PMSF 1 mM] was mixed with 300 mg of tissue powder and incubated for 10 min on ice with eventual vortex. Samples were sonicated for 10 min on an ice-cold water bath and filtered through one-layer miracloth. Proteins were quantified by Bradford assay with Bio-Rad protein assay (Bio-Rad, 5000001) and 10 μg of protein were loaded into SDS-PAGE gels. For ATG8 and NBR1 detection, 15 or 8% acrylamide/bis-acrylamide resolving gels were used, respectively. Proteins were transfered onto polyvinylidene difluoride membranes (Merck-Millipore). AntiATG8 raised against *Chlamydomonas reinhardtii* (Agrisera, AS14 2769) and antiNBR1 raised against *Arabidopsis thaliana* NBR1 (Agrisera, AS14 2805) were used at 1:2,000 dilution. Secondary antibody was used at 1:5,000 dilution. Blots were developed using ECL substrate (Thermo Fisher Scientific). The developing reaction was detected using a Chemidoc XRS+ (Bio-Rad). Band quantification was performed with Image Lab software (Bio-rad).

### Quantitative Real-Time PCR

Pooled de-achened receptacles of the different developmental stages or individual de-achened receptacles, in the case of the silencing experiment, were grounded in liquid nitrogen and RNA was extracted using a rapid CTAB method previously described (Gambino et al., 2008). RNA concentration was measured with a Nanodrop instrument (Thermo Fisher Scientific, USA) and samples were treated with DNAse TURBO (Thermo Fisher Scientific, AM2238) according to the manufacturer's protocol. 1 μg RNA was used for cDNA synthesis using iScript cDNA synthesis kit (Bio-Rad, 1708890) and 1/10 of each reverse transcription reaction was used for quantitative real-time PCR (qRT-PCR) analysis. SsoFast Evagreen supermix (Bio-Rad, USA, 1725200) was used for qRT-PCR. Reactions were run in a CFX Real Time PCR detection system (Bio-Rad) with the following protocol: 95°C, 10 min, 40 cycles of 95°C, 15 s and 60°, 1 min, 95°C, 1 min. qRT-PCR efficiency was determined by LinReg PCR program. Normalization was performed against *FaActin* and *FaDBP* genes (Galli et al., 2015) and ΔΔCT was used to quantify fold change difference expression among samples. All primers used in this experiment were designed with Primer3 software (primer3.ut.ee, Untergasser et al., 2012) and are listed in Supplementary Table 1-S1.

### Plasmid Construct, Transient Silencing, and 3-Methyladenine Infiltration Experiments

Infiltration experiments on strawberry receptacles were performed in *F. x ananassa* cv “San Andreas.” For transient silencing of *FxaATG5* or *FxaATG7*, RNAi constructs were developed using the gateway-based vector pHELLSGATE12 (Helliwell and Waterhouse, 2005). Forward and reverse specific primers were designed and attb1 and attb2 sites, respectively were added to them (Supplementary Table 1-S1). Sequences were PCR amplified using *F. x ananasa* cv. “San Andreas” cDNA as template. The resulting fragments (307 bp for *FxaATG5* and 358 bp for *FxaATG7*) were recombined in independent pDONR-Zeo. The corresponding pENTRY vectors were finally recombined with pHELLSGATE12. The design of this vector allows the recombination of the sequence of interest both in sense and antisense. AGL0 strain of *Agrobacterium tumefaciens* was transformed with the different constructs (pHELLSGATE12, PHELLSGATE12-FxaATG5i, and pHELLSGATE12-FxaATG7i) and grown at 28°C until OD_600_ reached 0,7. Cells were centrifuged at 3,000*g* for 10 min, resuspended in infiltration buffer (10 mM MgCl_2_, 10 mM MES, pH 5.6, and 200 mM acetosyringone), and incubated for at least 1 h at room temperature. Approximately 500 μl of *Agrobacterium* suspension was evenly infiltrated into green stage fruits (14 days after anthesis approximately) with the help of a 1 ml syringe. 14 fruits were injected with each construct. Fruits remained attached to the plant and were harvested, and flash-frozen in liquid nitrogen 9 days after infiltration. Achenes were removed from frozen fruits with the help of a scalpel. For 3-methyladenine (3-MA) treatment, infiltration assays were performed as described for silencing experiments. Green stage fruits were evenly injected either with mock (milli-Q water) or 5 mM solution of 3-MA (Merck, M9281) dissolved in milli-Q water. Fruits were harvested and photographs were taken 9 days after infiltration. 10 fruits were injected with either mock or 3-MA. Fruit size comparison was performed measuring fruit area in photographs using ImageJ.

## Results

### Autophagy Related Genes Are Differentially Expressed During Strawberry Fruit Ripening

The cultivated strawberry *F. x ananassa* is an allo-octoploid (2n = 8x = 56) originated by hybridization of two octoploid species in the 18th century (Duchesne, 1766). A recent study has established that the ancestral parental diploid species of *F. x ananassa* were *Fragaria iinumae, Fragaria niponica, Fragaria viridis*, and *Fragaria vesca*, this last one being the subgenome that has increased its dominance over the other three along evolution (Edger et al., 2019). For this work, we first searched in the genome of *F. vesca* for putative orthologs of the *Arabidopsis* autophagy-related genes. As expected, we found orthologs of all key *ATG* genes, the selective autophagy gene *NBR1*, and the Phosphatidyl inositol 3 kinase type III VPS34 (Supplementary Table 1-S2). It is interesting to note that the number of genes belonging to each family in the diploid *F. vesca* was very similar to *Arabidopsis thaliana*, including those described as single copy genes.

Next, we identified in the recently sequenced and re-annotated genome of the octoploid *F. x ananassa* (Edger et al., 2019; Liu et al., 2021) the homoeologs for the autophagy-related genes found in *F. vesca*. Previously, our group generated a set of transcriptomic data during the fruit ripening of *F. x ananassa* after mapping the reads of a RNAseq experiment in the sequenced reference genome *F. vesca* (Sánchez-Sevilla et al., 2017). In the present work, we have mapped those reads to the last annotation of *F. x ananassa* genome and we have analyzed the expression of key autophagy genes belonging to the different functional groups of this pathway in the receptacle. Different autophagy genes participate at the different stages of the process. These stages are: (i) induction (ATG1, ATG13, ATG11, and ATG101), (ii) vesicle nucleation (ATG6 and VPS34), (iii) membrane delivery (ATG9, ATG2, and ATG18), and (iv) autophagosome expansion and closure (ATG4, ATG5, ATG7, ATG8, ATG10, and ATG12) (Marshall and Vierstra, 2018). We have found that all these autophagy genes are expressed during strawberry fruit receptacle ripening (Supplementary Table 2).

**Figure 1:**
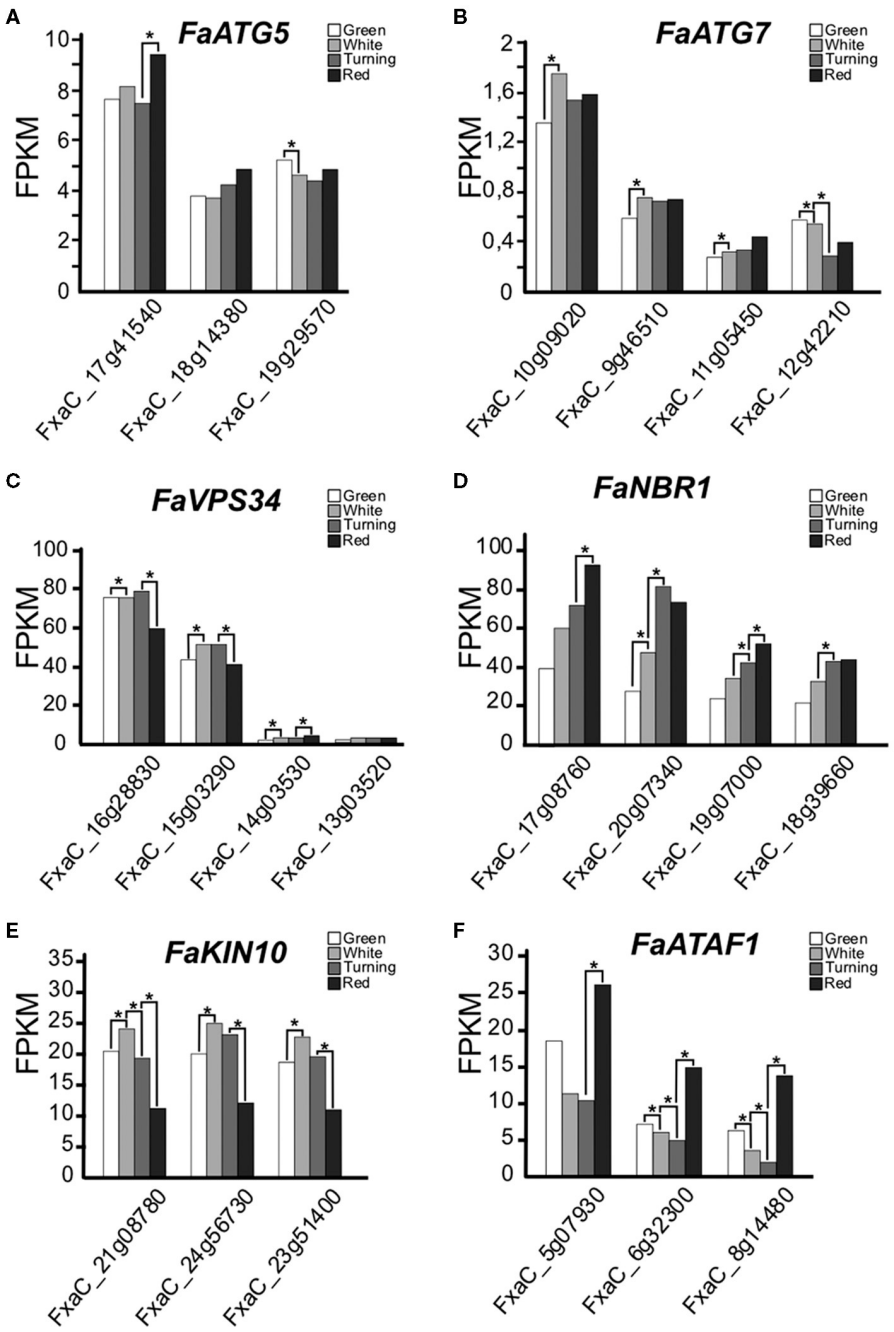
**(A–F)** Expression of autophagy-related genes along ripening in *F. x ananassa* cv. Camarosa. Data for this analysis were extracted from the RNAseq generated by Sánchez-Sevilla et al. (2017). RNA was extracted from de-achene receptacles at the stages green, white, turning, and red. Statistical significance was determined between one stage and the next; it is indicated with an asterisk. Statistical analysis was performed with the false discovery rate (FDR) method after Benjamini-Hochberg correction for multiple-testing. Complete statistical analysis can be found in Supplementary Table 2.

ATG5 and ATG7 are two proteins that are essential for the lipidation of ATG8, a process required for autophagosome expansion (Marshall and Vierstra, 2018). It has been reported that mutants of these genes show impaired autophagosomes formation and autophagy blocking in different plant species, mammals, and yeast (Phillips et al., 2008; Minina et al., 2013; Sanchez-Vera et al., 2017; Klionsky et al., 2021). We have identified three *ATG5* and four *ATG7* genes in *F. x ananassa*. In the ripening fruits, the three *ATG5* and two of the *ATG7* genes are expressed (above 0,5 FPKM), with minor differences, between the different developmental stages (Figures 1A,B).

VPS34 is a phosphatidyl inositol 3-kinase (PI3K) that belongs to the PI3K complex. This complex participates at early stages of the autophagy process with an essential role in vesicle nucleation (Zhuang et al., 2018). We have found four *VPS34* genes expressed during ripening in *F. x ananassa* showing significant expression increase in white and turning compared to green and red (Figure 1C).

NBR1 is a protein that acts as a cargo receptor and that gets degraded during autophagy. It has been proposed to play a conserved role in selective autophagy in plants (Svenning et al., 2011; Ji et al., 2020). We found that the four putative homoeologs of this gene were expressed, and their expression levels steadily increased from green to red fruits (Figure 1D).

Also in plants, two regulators of the autophagy process have been proposed: the transcription factor ATAF1 (Garapati et al., 2015) and the SnRK1 kinase complex (Soto-Burgos and Bassham, 2017). The expression of the *KIN10* genes, encoding the catalytic subunit of the complex, and the *ATAF1* genes in the ripening receptacle of the strawberry fruit are shown in Figures 1E,F. While the *KIN10* expression peaked at the white stage, the expression of *ATAF1* displayed higher values at green and red stages.

**Figure 2:**
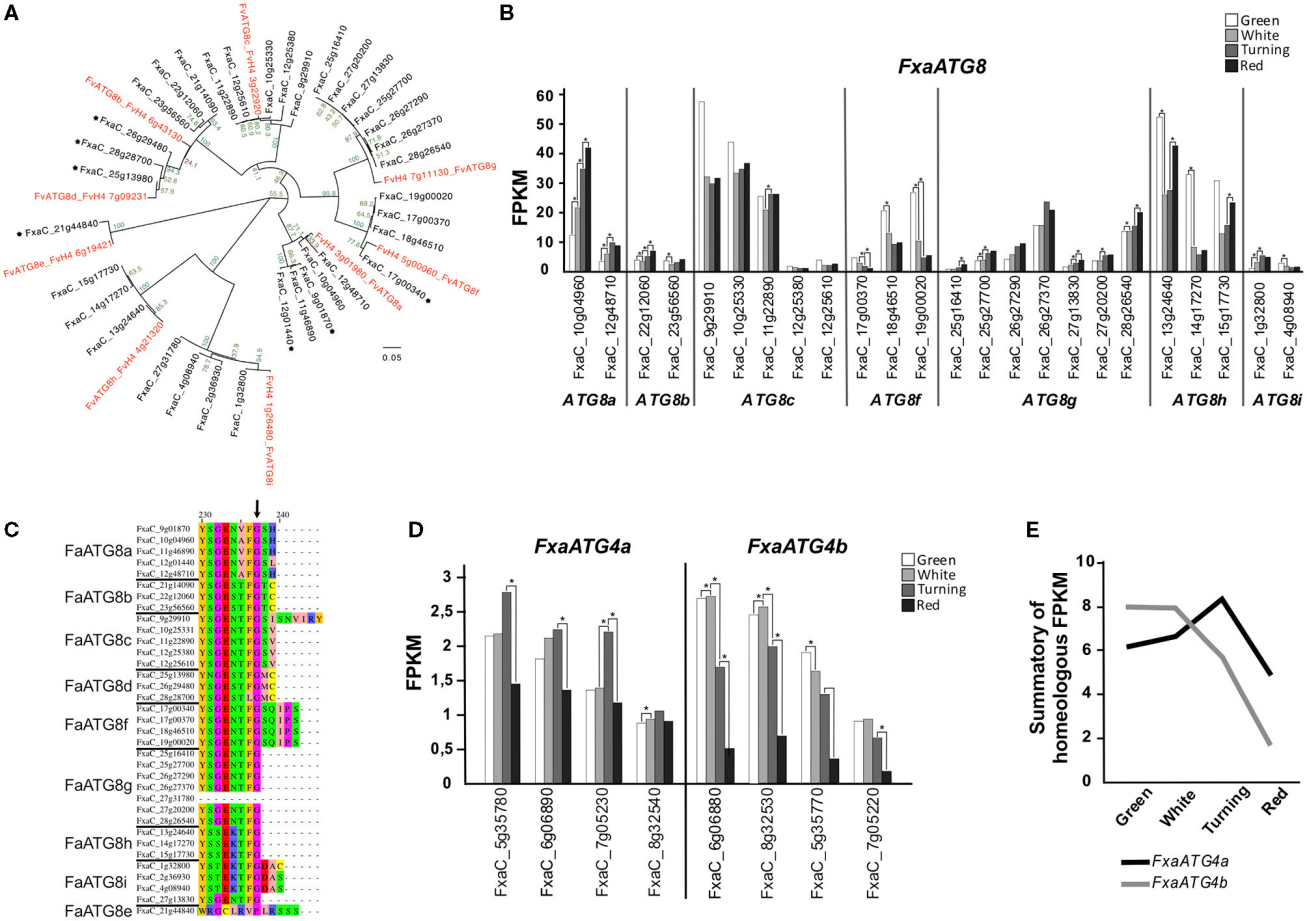
Analysis of FaATG8 and FaATG4. **(A)** Phylogenetic tree of *F. vesca* and *F. x ananassa ATG8* genes. Nucleotide sequences were aligned by MAFFT and the tree was generated by the neighbor-joining method and displayed using FigTree. Asterisks mark genes with <0,5 FPKM expression values in all tissue tested (achenes, receptacle, leaves, and roots). **(B)** Representation of Fa*ATG8* genes expressed at least 1 FPKM in any stage of the process of receptacle ripening. **(C)** Alignment of C′-terminus end of the different *FaATG8* homoeologs. Arrow indicates the conserved Glycine essential for lipidation. **(D)** Representation of *FaATG4* expression during strawberry ripening. **(E)** Expression trend of *FaATG4a* and *FaATG4b* homoeologs during ripening. Data shown in **(B–E)** were extracted from the RNAseq dataset generated by Sánchez-Sevilla et al. (2017). Statistical significance between one stage and the following stage it is indicated with an asterisk. Statistical analysis was performed with the false discovery rate (FDR) method after Benjamini-Hochberg correction for multiple-testing. Complete statistical analysis can be found in Supplementary Table 2. Alignment that generated phylogenetic tree (2A) can be found in Supplementary Data Sheet 1.

ATG8 proteins play a key role in the autophagosome expansion that occurs during autophagy (Martens and Fracchiolla, 2020). Upon autophagy activation, ATG8 undergoes a lipidation process mediated by a ubiquitin-like system in which ATG5 and ATG7 participate (Marshall and Vierstra, 2018). This lipidation consists of the binding of a phosphatidylethanolamine (PE) molecule to a Glycine at the ATG8 C-terminus end. For this to happen, first ATG8 has to be cleaved by ATG4 in order to get this Glycine exposed (Yoshimoto et al., 2004). Once ATG8 proteins are lipidated, they can be anchored to the autophagosome membrane. We found nine *ATG8* genes in the genome of *F. vesca* and 36 homoeologs in *F. x ananassa*. To establish the relation between each *F. vesca* gene with its correspondent homoeologs, we performed an alignment of the nucleotide sequences with the *F. vesca* and *F. x ananassa ATG8* genes that produced the dendrogram displayed in Figure 2A. The clustering pattern indicates the correspondences between the genes of both species. Some of the *ATG8* homoeologs identified in the *F. x ananassa* genome did not show expression in any of the tissues analyzed (receptacle, achenes, leaves, and roots) (indicated with asterisks in Figure 2A) and two of them were expressed <1 FPKM during receptacle ripening, so they were not included in the expression analysis. The expression of the *ATG8* genes at four stages of receptacle ripening (green, white, turning, and red) is shown in Figure 2B. There is a high variability in the expression both in absolute values and stage-dependent values. Three main expression patterns were found, one with increasing expression from green to red stages (*ATG8a, ATG8g*), another decreasing from green to red (*ATG8f* ), and a third with highest values at green and red stages (*ATG8b, ATG8c, ATG8h*).

ATG8 proteins are also essential for autophagosome-cargo recognition. ATG8 present two hydrophobic pockets that determine the interaction with proteins containing the so called ATG8-interaction motifs (or AIM) (Noda et al., 2010). These AIM-containing proteins are located at the surface of organelles, protein complexes, and other structures working as docking platforms for ATG8 and around which the autophagosome grows and engulfs them (Marshall and Vierstra, 2018). When analyzing the FaATG8 aminoacidic sequences, we found that the residues located at the key positions of the two hydrophobic pockets shows a high level of conservation (Supplementary Figure 1, black and red arrows) (Kellner et al., 2017). Furthermore, apart from two of them, all shared the C-terminal Glycine required for the lipidation (Figure 2C), however the downstream sequence showed high variability both in terms of amino acid number (0, 2, 3, and 5) and nature.

Processing of ATG8 previous to its lipidation is done by the protease ATG4. In the diploid *F. vesca*, as reported in *Arabidopsis*, we identified two *ATG4* genes, while in *F. ananassa* eight ATG4 genes were identified included in two possibly homoeologs groups, *ATG4a* and *ATG4b*. Their expression is shown in Figure 2D. The analyses of the joint expression of all putative homoeologs of *ATG4a* and *ATG4b* (Figure 2E) show that from green to white stage *ATG4b* has a prevalent expression and goes down from white to red, whereas *ATG4a* expression increases from white to turning and gets reduced toward red.

### Autophagic Structures Are Present at Different Stages of Strawberry Receptacle Ripening

**Figure 3:**
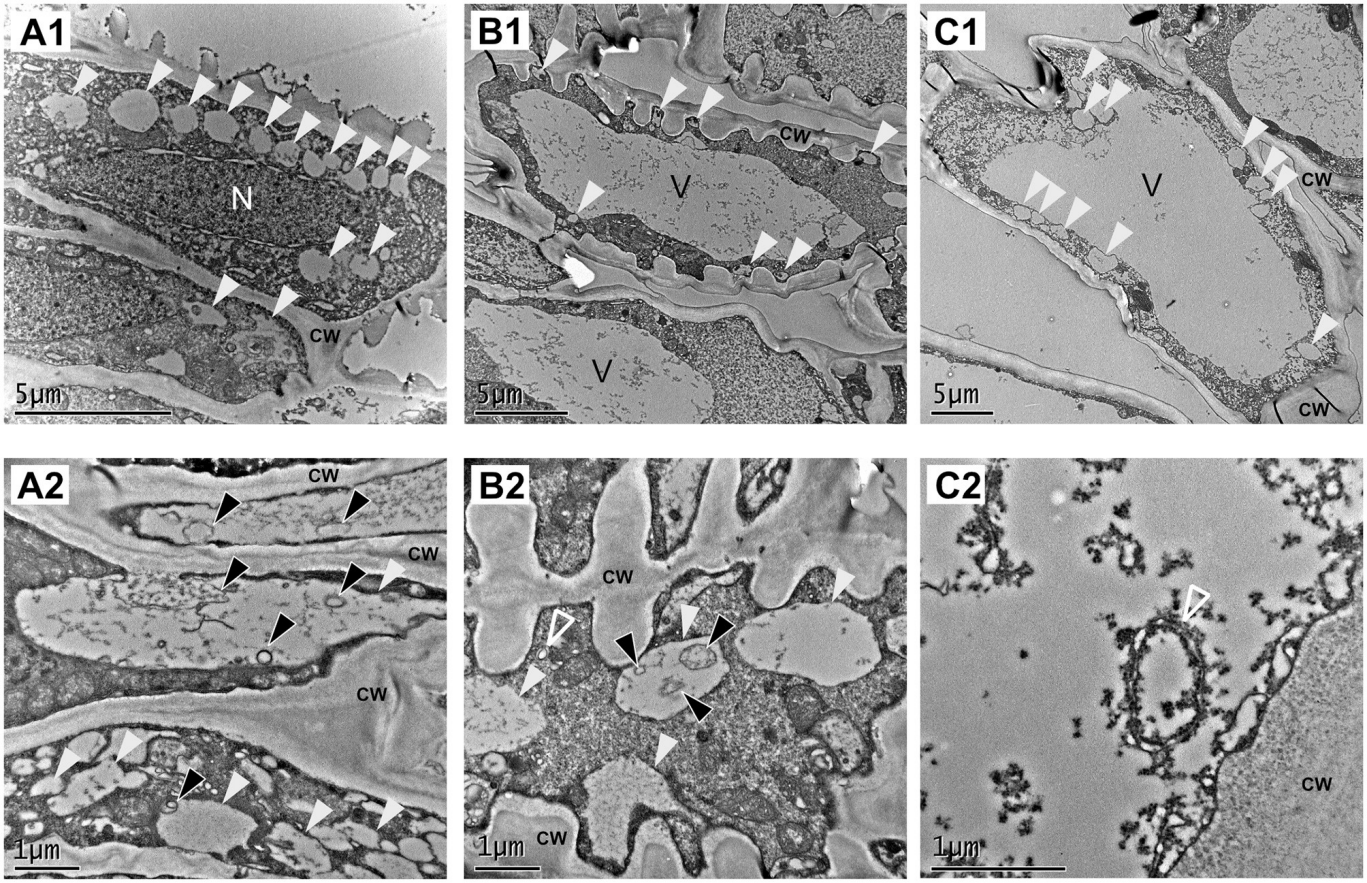
Vascular tissue is a place of active autophagy. TEM micrographs from representative cells of the green receptacle vascular tissue. **(A**–**C)** Different cell types found in vascular tissue. **(A,C)** Parenchymatic cells of the vascular tissue. **(B)** Immature xylem cells. **(1)** Overview of the cells and **(2)** close up of the same type of cells showing autophagy-related structures. White arrowheads: single membrane compartments with degraded content, black arrowheads: autophagic bodies, empty white arrowheads: autophagosomes. CW, cell wall; n, nucleus; v, Vacuole.

The transcriptomic data indicate that genes involved in autophagy are expressed during fruit ripening. To investigate the presence of autophagic process at the cellular level, we performed an ultrastructure analysis of the receptacle along fruit ripening using transmission electron microscopy (TEM). It has been described that autophagy is involved in the process of tracheary element formation (Kwon et al., 2010), so we analyzed cells at the vascular tissue from green receptacle. The strawberry receptacle develops vascular bundles that connect the achenes with the pith (Aharoni et al., 2002). As expected, a significantly high amount of autophagy-related structures could be found in immature xylem cells (Figure 3B1). These cells are easily distinguishable in TEM images thanks to the characteristic patterned cell wall deposition that they present. Many single membrane vesicles (of around 0,5 to 1 micrometers) previously described as autolysosome-like structures (Takatsuka et al., 2004; Sanchez-Vera et al., 2017), presenting electron - dense amorphous content in some cases or structures resembling autophagic bodies in other cases, were found in those cells and in the surrounding cells, probably xylem parenchymatic cells (Figures 3A1,A2,B1,B2,C1 white and black arrowheads, respectively). Autophagosomes were also visible in both cell types (Figures 3B2,C2, empty white arrowheads).

**Figure 4:**
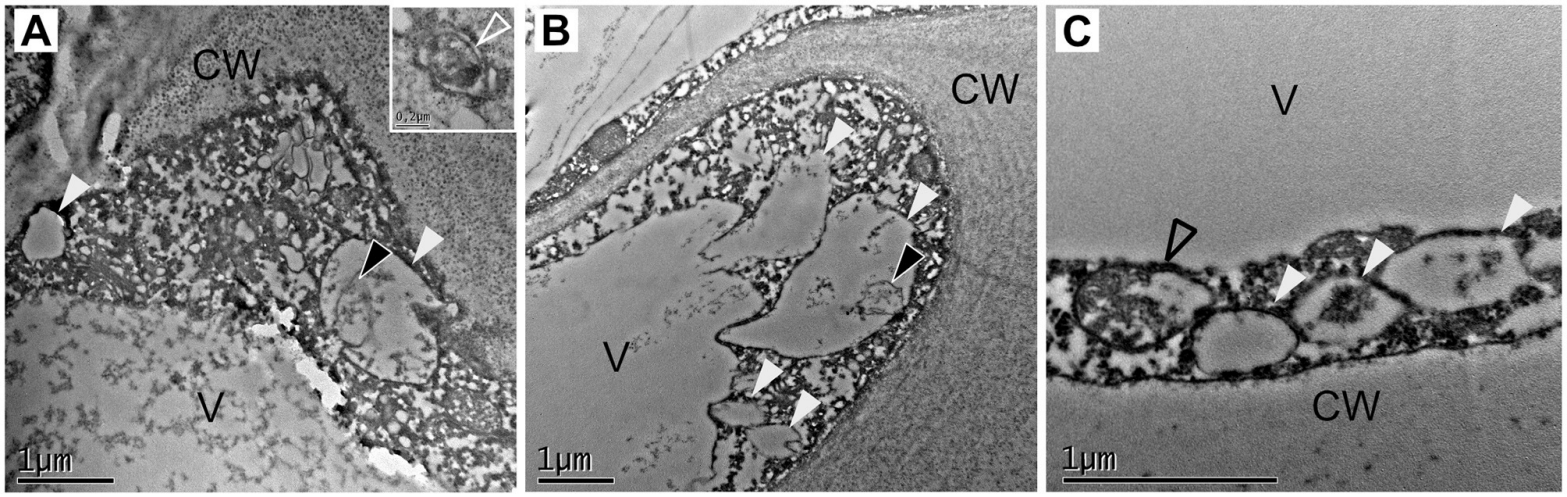
Identification of autophagy-related structures in *F x ananassa* receptacle along ripening. TEM micrographs from receptacle cortical cells showing autophagy-related structures. **(A)** Green receptacle cell, **(B)** white receptacle cell, and **(C)** red receptacle cell. White arrowheads: single membrane compartments with degraded content, black arrowheads: autophagic bodies, empty black arrowhead: disassembling mitochondria. CW, cell wall; V, Vacuole.

Cells present at the cortex and pith of *F x ananassa* receptacle are composed by a big central vacuole and a thin layer of cytoplasm pressed to the cell wall. When we analyzed cortical cells located at the subepidermal space of the receptacle of *F. x ananassa* cv. “Camarosa” at stages green, white, and red both autophagosomes (Figure 4A, inlet) and autolysosome-like structures with degrading content and autophagic bodies were found (Figures 4A–C; white and black arrowheads, respectively). This made them the most abundant the autolysosome-like structures. It is interesting to note that even though autophagy is considered a relatively fast process in plants, difficult in some cases to monitor by TEM without the help of inhibitors, we have been able to identify those structures directly in non-treated cells.

### Strawberry Fruits Show Two Waves of Increased Autophagy Flux During Ripening

**Figure 5:**
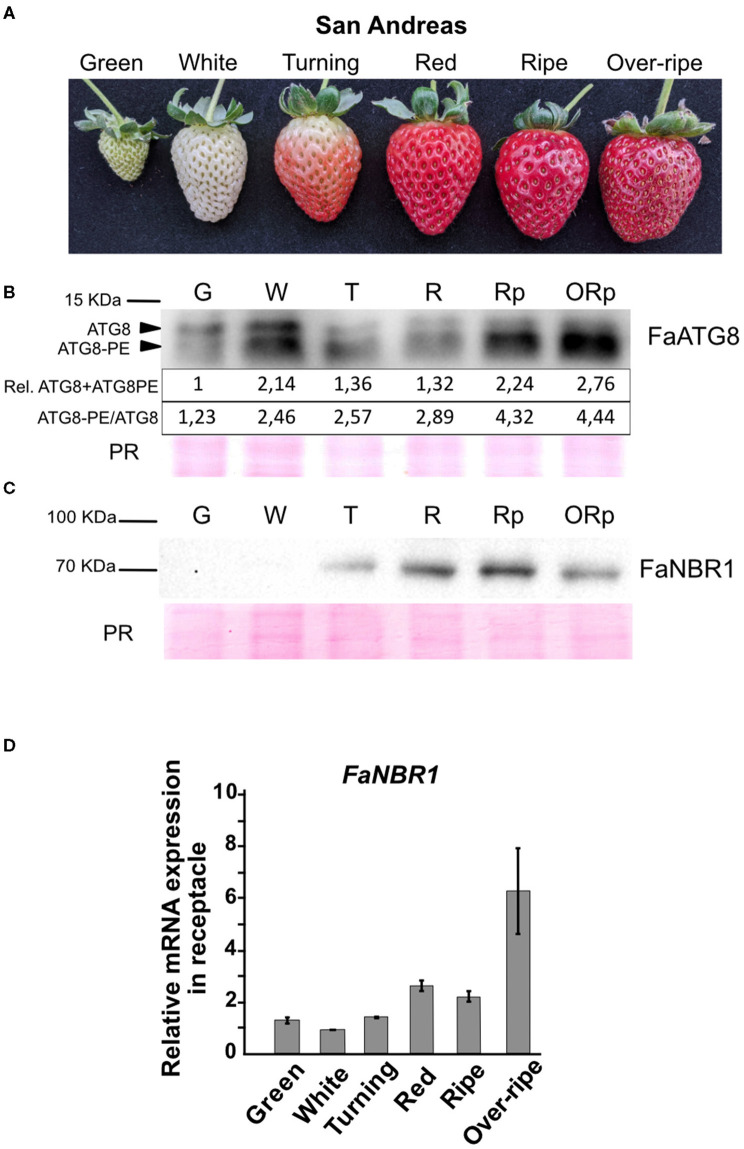
Identification of two waves of autophagy flux along strawberry ripening. **(A)** Stages of strawberry ripening (cv. “San Andreas”) analyzed for autophagy flux. **(B)** Immunoblot of ATG8 showing non-lipidated (ATG8) and lipidated form (ATG8-PE) along ripening. Quantifications of band intensities are shown in arbitrary units. Rel. ATG8+ATG8PE shows relative quantification of the summatory of ATG8 and ATG8PE signal. ATG8PE/ATG8 shows the ratio of both protein forms. **(C)** Immunoblot of NBR1 along ripening. **(D)** qRT-PCR analysis of *FaNBR1* in *F. x ananassa* cv “San Andreas” along ripening. Error bars represent standard deviation. PR, Ponceau red.

Although the differential expression of autophagy-related genes and the presence of autophagy structures at the cellular level supports that autophagy could be active during ripening, this observation does not demonstrate by itself the existence of an increased autophagy flux. Therefore, we analyzed the levels of ATG8 lipidation and NBR1 that have been described as markers of autophagy flux (Klionsky et al., 2021). As previously indicated, lipidation of ATG8 is essential for autophagosome formation. When ATG8 lipidation is abolished, as happens in certain *ATG* gene mutants, autophagosome formation is prevented and autophagy flux is blocked (Chung et al., 2010). Thus, the level of ATG8 lipidation has been used as a measure of autophagy activity (Bao et al., 2017). Therefore, we examined by immunoblot analysis the levels of ATG8 protein and its lipidated form (ATG8-PE) using an antibody raised against *Chlamydomonas rheinardtii* ATG8 that recognizes all ATG8 isoforms (Pérez-Pérez et al., 2010). For this analysis we decided to include two more developmental stages corresponding with the late maturation process and to use the variety “San Andreas” that is a day neutral cultivar. “San Andreas” comes from the same breeding program of cv. “Camarosa” but presented slightly lower content of anthocyanins and phenolics at the ripe stage, as well as lower fruit firmness, under specific growth conditions (Lalk et al., 2020), and has the advantage of producing fruits throughout the year. Thus, we analyzed the receptacle of “San Andreas” strawberry fruits at six developmental stages: green, white, turning, red, ripe, and over-ripe (pre-senescent) fruits (Figure 5A). The relative level of total ATG8 protein, which is the sum of non-lipidated ATG8 and lipidated ATG8, showed a first maxima at the white stage and a second maxima at the end of the ripening period (Figure 5B). Interestingly, the ratio ATG8PE/ATG8 shows an important increase at the white stage and a more dramatic one at the ripe stage (1,75-fold change of the ratio between white and ripe). These results clearly show that there is an autophagy induction at both stages supported both by an increase in the ATG8 protein amount and by its lipidation level, being the second induction of higher intensity. As expected, cv “Camarosa” shows a very similar pattern of ATG8 expression and lipidation at those stages (Supplementary Figure 2B).

NBR1 is an autophagy substrate, therefore when autophagy is active NBR1 gets degraded, so its protein levels gets reduced even though its mRNA expression remains high. Regarding the levels of NBR1, we found that the protein levels at the over-ripe stage was lower than at the ripe stage (Figure 5C). To elucidate whether this was an effect of the degradation through autophagy of NBR1 or a diminishing of the mRNA expression, we performed qRT-PCR of *NBR1* in the receptacle at the different stages (Figure 5D). Our data show that *NBR1* mRNA levels are not reduced at the over-ripe stage, so our results support that the depletion of NBR1 protein levels at the over-ripe stage is probably due to its degradation by autophagy, consistent with the levels of ATG8 lipidation. It is interesting to note that we see a correlation between lipidation levels of ATG8 at the white stage and depletion of NBR1 protein but not mRNA also in “Camarosa” (Supplementary Figures 2B,C).

Taken all together, our data support the presence of two marked waves of autophagy flux induction during strawberry ripening supported by the ratio of ATG8-PE/ATG8 and the level of both protein forms, peaking at white and ripe stages.

### Autophagy Inhibition Affects the Fruit Development and Delays the Ripening

**Figure 6:**
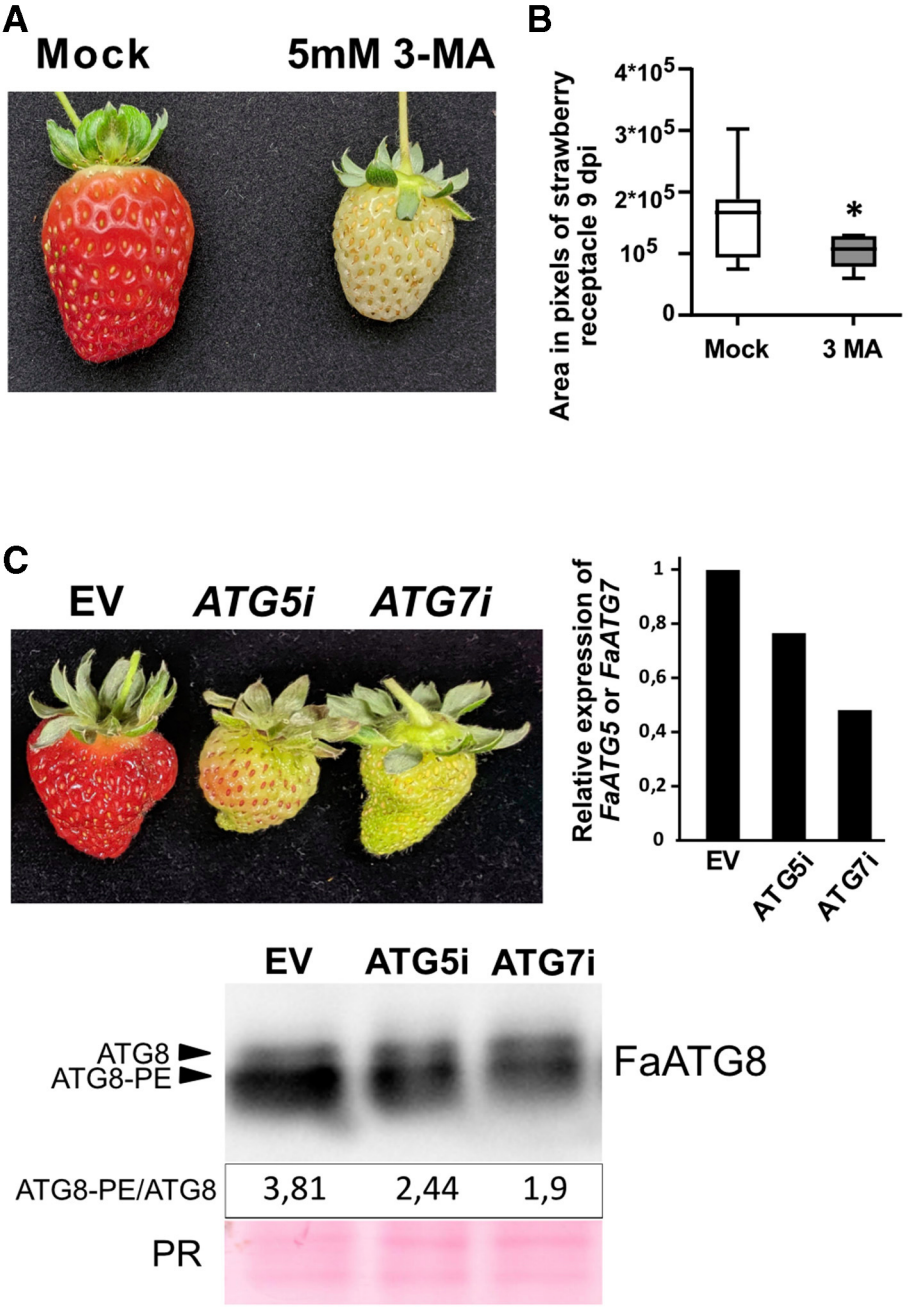
Effect of autophagy blocking over strawberry ripening. **(A)** Representative image showing the effect over ripening of 3-MA in *F x ananassa* cv “San Andreas”. **(B)** Fruit area in 3-MA treated fruits compared to mock treated samples. Variance difference between mock treated lines and 3-MA treated lines were analyzed using an F-test and * denote significantly different with a *P* < 0,005. **(C)** Up-left, representative image showing the effect over ripening of *FaATG5* or *FaATG7* silencing. Up-right, qRT-PCR analysis of *ATG5* or *ATG7* expression in infiltrated receptacles. Down, immunoblot analysis of ATG8 levels in control and silenced fruits and quantification of ratio between lipidated (ATG8-PE) and non-lipidated form (ATG8). The **(C)** panel is representative of three independent experiments. EV, Empty vector; PR, Ponceau Red.

A number of autophagy inhibitors have been reported (Marshall and Vierstra, 2018). Among them, the 3-Methyladenine (3-MA) has been broadly used to block autophagy in different species including plants (Klionsky et al., 2021). 3-MA inhibits the Phosphatidylinositol-3-kinase activity of the PI3K complex (Takatsuka et al., 2004), more precisely through inhibition of VPS34, a protein highly expressed throughout ripening (Figure 1C) that participates in the vesicle nucleation stage of the autophagy pathway. Treatment of “San Andreas” fruits at the green stage with a 5 mM solution of 3-MA caused a dramatic delay in the ripening progress (Figure 6A) compared with the mock treated fruits. The delay in ripening was evident by the lack of red color, probably due to the lack of anthocyanin accumulation, and by the reduced size of the fruit. Although the variability in fruit size in the water infiltrated fruits was high, we observed a significant reduction in the size range that 3-MA treated fruits reached 9 days after infiltration (Figure 6B).

It is possible that besides its effect over autophagy, inhibition of VPS34 by 3-MA could be having an additional effect over other cellular processes different from autophagy, such as the endocytic pathway where VPS34 also participates (Bhati et al., 2021). Therefore, we performed transient silencing in green fruits of two of the genes essential for the ATG8 lipidation process, *ATG5* and *ATG7*, using RNAi. The phenotypic analysis of the infiltrated fruits showed a significant delay of the fruit ripening in the silenced fruits compared with the controls (Figure 6C). Next, we were interested in determining whether this silencing could have a correlation with a diminishing in the lipidation of ATG8. Thus, we measured the ATG8 levels by immunoblot as described before and quantified the lipidation to non-lipidation ratio. As expected, the ratio of ATG8-PE/ATG8 also decreased in the transiently silenced fruits confirming that the reduction of ATG5 or ATG7 activity was having an effect over ATG8 lipidation process (Figure 6C).

Overall, our transcriptomic data, cytological analyses, and biochemical and genetic studies supports that autophagy has a relevant role in the ripening of the strawberry fruit, happening in two waves and with direct implications in, at least, the vascular development and the senescence of the fruit.

## Discussion

Our study shows that strawberry receptacle is a place of constant autophagy activity with two points of autophagy flux induction. Analysis of *ATG* genes and ATG8 protein expression in the receptacle of strawberry fruits from green to red stage shows a basal level of transcripts at every stage, with enhanced expression of some of them at specific stages. On the other hand, autophagy related structures can be observed in the cortical cells in different stages of the ripening process. This supports a dual role of autophagy during strawberry fruit ripening, as a housekeeping process to maintain the cellular nutrient homeostasis and as a specific recycling mechanism to cope with developmental conditions that requires the generation of new cellular compounds or environmental situations that challenge the energy balance in the cell (McLoughlin et al., 2020).

A process that accompanies strawberry fruit growth from the early stages is the formation of the vascular network that connects the achenes to the different cell types of the receptacle and the central pith. Qualitative markers of vascular development were found at all the developmental stages of strawberry ripening (Aharoni et al., 2002). In other plant organs, the activation of autophagy in the differentiation of the xylem before the lignification of the cells has been reported (Kwon et al., 2010; Wojciechowska et al., 2019). Here we show the presence of autolysosome-like structures and autophagosomes in cells of the vascular tissue of the receptacle, so it would be expected that occurrence of autophagy in the fruits is taking place associated with the vascular development, that is parallel to the growth in size of the fruit. This would also explain the relatively steady expression of some of the *ATG* genes found in the growing receptacle, from green to red stage.

The occurrence of an elevated number of ATG8 isoforms in plants opens the question of their contribution to the diversification of selective autophagy pathways (Kellner et al., 2017). It has been proposed that possible functional diversification can be supported by their association with a number of ATG8-interacting proteins which act as cargo receptors (Marshall and Vierstra, 2018). Sequence analysis of the *ATG8* genes in the *F. x ananassa* genome shows high conservation of residues in the two hydrophobic pockets that interact with proteins containing the AIM domain (Kellner et al., 2017). There are some differences in the amino acid positions around the binding pockets in the ATG8 proteins, but only the structural analysis of the ATG8 isoforms and the possible interactors could establish the relevance of the sequence variability on their functional diversity. However, the occurrence of co-expression patterns between the ATG8 isoforms and the possible interacting proteins would be a step to explain the functional diversity of ATG8. In this sense, we have found that the known NBR1 interactor (Svenning et al., 2011) presents the highest expression level at late stages of fruit ripening, coincident with the peak of expression of several of the ATG8 isoforms. Previously, it has been reported that the NBR1-dependent selective autophagy in plants occurs under different stress and non-stress conditions (Zhou et al., 2013; Chi et al., 2020; Jung et al., 2020), being common to the different conditions observed during the occurrence of oxidative stress. An early analysis of the transcriptional changes in the ripening strawberry fruit associated the ripening process to a response to oxidative stress (Aharoni et al., 2002), which would be in agreement with the possible interaction of some isoforms of the ATG8 family and NBR1 in the autophagy occurring in the ripening receptacle of the strawberry fruit. In *Arabidopsis*, NBR1 showed different levels of interaction with the members of the ATG8 family of proteins (Svenning et al., 2011).

The set of putative homoeologs of *ATG8c, ATG8f, and ATG8h* genes present an expression induction in green. This suggests a specific involvement in the autophagic processes occurring at this early stage. Among them, in *ATG8c* and *ATG8f*, the increase of expression happens only in green but not in other stages of the ripening process. Intriguingly, all members of the ATG8f and one of the ATG8c show a longer amino acid sequence at the C-terminus after the site of proteolysis by ATG4. In the diploid *F. vesca*, as in *Arabidopsis* (Seo et al., 2016), two *ATG4* and nine *ATG8* genes are identified. In *Arabidopsis, in vitro* studies of the activity of the two ATG4 against different ATG8 showed a difference in the activity and the substrate specificity of the two proteases (Woo et al., 2014). Moreover, broad studies on the specificity between ATG4 and ATG8 in a more diverse range of species support that the processing activity is determined by the ATG8 sequence rather than the ATG4 (Seo et al., 2016). Interestingly, the joint expression of all the *F. x ananassa* ATG4 genes corresponding to the two *F. vesca* genes show equivalent values of expression, but with ATG4a having the highest expression at the turning stage and ATG4b at the green/white stages. Whether the C-terminal sequences of the strawberry ATG8s determine the selectivity for its processing by ATG4 would be of interest to know, since it would provide valuable information about the functional diversity of the ATG8 genes in strawberry.

Our analysis of the ATG8 lipidation, which is a critical step in the autophagy progress, points to two time points during ripening of highest ATG8 content and lipidation: during the white stage and at the end of ripening in a pre-senescent stage. Growth of strawberry fruit, as in other fleshy fruits, is supported by fruit metabolism that must be energetically adjusted during the transition from green to ripe stage (Carrari and Fernie, 2006; Fait et al., 2008; Jing and Malladi, 2020; Martín-Pizarro et al., 2021). In this process, there is a critical step when the limited photosynthetic capacity of the green fruit is completely lost after the loss of chlorophyll. Autophagy has been associated with the metabolic adjustment taking place during the plant developmental transitions (Michaeli et al., 2016). The increase of ATG8 lipidation found in the receptacle at the white stage can be explained by the need to maintain the nutrients supply required to continue the fruit growth. The growth conditions of the fruit under the loss of chlorophyll could be similar to the nutrient deprivation found in other plant tissues, where the autophagic response contributes to the energy adjustment (McLoughlin et al., 2020). The SnRK1 complex is a kinase that functions as an energy sensor (Broeckx et al., 2016) and acts as an autophagy activator (Marshall and Vierstra, 2018). It is significant that the expression of KIN10, a component of the SnRK1 complex, increases from green to white stage in the three homoeologs. It would be of interest to know whether KIN10 also plays a regulatory role of autophagy in the receptacle of the strawberry fruit, that increases the autophagic flux at the white stage as a response to low energy conditions, as reported in *Arabidopsis* (Soto-Burgos and Bassham, 2017). In climacteric fruits the reduction of photosynthetic capacity during ripening is coupled with the burst of aerobic metabolism, whose energy balance is more favorable. To our knowledge, the only publications showing increasing expression of autophagy genes with ripening has been made in non-climacteric fruits such as pepper, grapevine, and the present one made in strawberry (Ghan et al., 2017; López-Vidal et al., 2020). This brings the questions of whether autophagy is a relevant process during ripening of climacteric fruits.

Several autophagy inhibitors have been described in plants although most of them have effects over other non-autophagy processes (Marshall and Vierstra, 2018). 3-Methyladenine is a compound that inhibits VPS34 of the PI3K complex that participates in autophagy initiation but also in the endocytic pathway (Bhati et al., 2021). The effect over ripening of 3-MA treatment is very similar to the effect of *ATG5* or *ATG7* silencing, this is a delay in the progress of the process in both cases, showing clearly that autophagy has an important role in the ripening of strawberry. Regarding the possible implications of the endocytic pathway during strawberry ripening a more detailed study, treating fruits with specific endocytic pathway inhibitors such a Brefeldin A, could shed light into this question.

Aging and senescence are physiological stages where autophagy also plays a main role in plants (Wang and Schippers, 2019). The final stage of fruit ripening is followed by senescence. This is a phase of development that it is dependent on cell viability and the expression of specific genes (Thomas, 2013). This process is a way to recycle resources from aging organs to developing structures. We have found that the abundance of ATG8-PE greatly increases with fruit ripening, reaching a highest value in over-ripe fruits. In addition, the increased expression of cargo receptor *NBR1* with ripening and over-ripening and the decreased content of the NBR1 protein in the over-ripe fruits is further proof of the induction of autophagy at these developmental stages. Our results support that autophagy continues after fruit ripening as a recycling strategy prior to the onset of senescence. Analysis in *Arabidopsis* of autophagy function, by the induction of ATG genes expression or silencing in mutant lines under aging and senescence, concluded the existence of a dual role for autophagy, that prevents senescing while aging, but controls the salvage pathway when senescence occurs. The possible role of autophagy to prevent senescence in strawberry fruits could be critical for conservation of the fruit integrity after harvest since post-harvest behavior of the fruits mimics a senescent process (Pott et al., 2020).

Our results in strawberry point to a house-keeping autophagy activity along fruit growth inherent to growth processes as vascular development, as well as two periods of enhanced autophagy in response to nutrients shortage and senescence. To our knowledge, this is the first work showing clear evidence of the role of autophagy in the process of fleshy fruit ripening. The knowledge of the components and the extension of autophagy in the growth and development of fleshy fruits might be of great relevance in a sink organ that is predominantly heterotrophic.

## Data Availability Statement

Publicly available datasets were analyzed in this study. This data can be found here: https://www.ebi.ac.uk/ena/browser/view/PRJEB12420.

## Author Contributions

VS-V and VV conceived the study. VS-V, MB, and VV planned and reviewed the experiments. JS-S identified the *F. x ananassa* genes and re-mapped and quantified the RNAseq data. VS-V executed the experiments, analyzed the data, and wrote the paper. All authors reviewed and approved the final manuscript.

## Conflict of Interest

The authors declare that the research was conducted in the absence of any commercial or financial relationships that could be construed as a potential conflict of interest.

## Publisher's Note

All claims expressed in this article are solely those of the authors and do not necessarily represent those of their affiliated organizations, or those of the publisher, the editors and the reviewers. Any product that may be evaluated in this article, or claim that may be made by its manufacturer, is not guaranteed or endorsed by the publisher.

## Supplementary Material

Supplementary Figure 1 Alignment of FaATG8 aminoacidic sequences. Black and red arrows mark the residues that form the W and L pockets as shown for ATG8 from Brassicales, Solanaceae, and Poaceae (Kellner et al., 2017). Black asterisk marks the conserved Glycine to which PE is bond after ATG8 lipidation.

Supplementary Figure 2 Analysis of autophagy flux in *F. x ananassa* cv. “Camarosa,” **(A)** Stages of strawberry ripening in *F. x ananassa* cv. “Camarosa” analyzed for autophagy flux. **(B)** Immunoblot of ATG8 showing non-lipidated (ATG8) and lipidated form (ATG8-PE) and immunoblot of NBR1 along ripening. **(C)** Summatory of all NBR1 homoeologs RNAseq data.

Supplementary Data Sheet 1 Alignment of the *FaATG8* nucleotides sequences that generated phylogenetic tree shown in Figure 2A.

Supplementary Table 1 **(S1)** List of primers used in this work. **(S2)** Autophagy related genes found in *F. vesca* genome.

Supplementary Table 2 Expression levels in FPKM of all autophagy-related genes analyzed in this work along ripening in *F. x ananassa* receptacle and achenes.
