# Supplementary figures and images for: Autophagy Is Required for Strawberry Fruit Ripening
Source: Front Plant Sci. 2021 Aug 27;12:688481. doi: 10.3389/fpls.2021.688481 (PMC8429490; doi:10.3389/fpls.2021.688481)

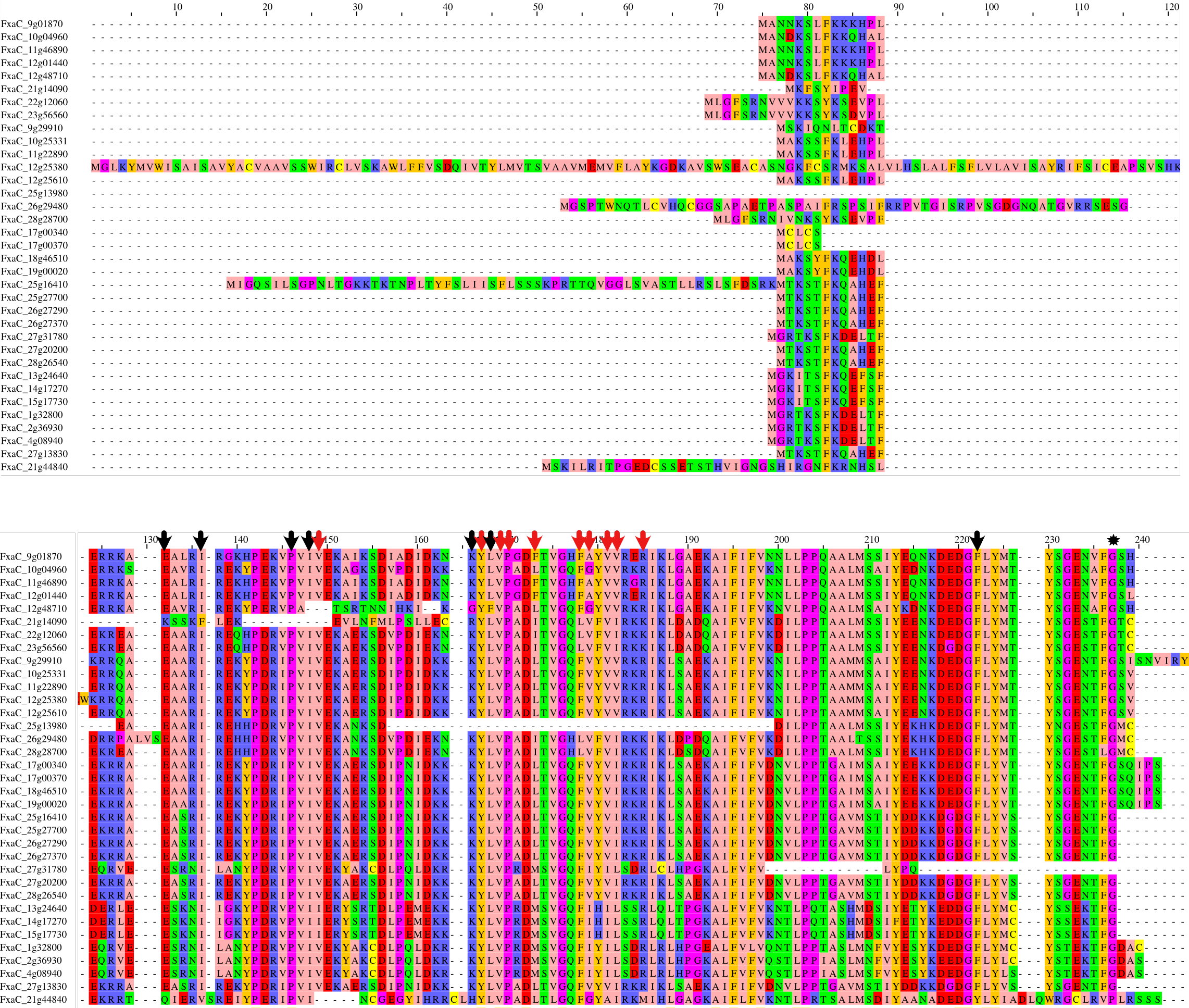

Supplement: Supplementary Figure 1 — Alignment of FaATG8 aminoacidic sequences. Black and red arrows mark the residues that form the W and L pockets as shown for ATG8 from Brassicales, Solanaceae, and Poaceae (Kellner et al., 2017). Black asterisk marks the conserved Glycine to which PE is bond after ATG8 lipidation. [file Image_1.TIFF]

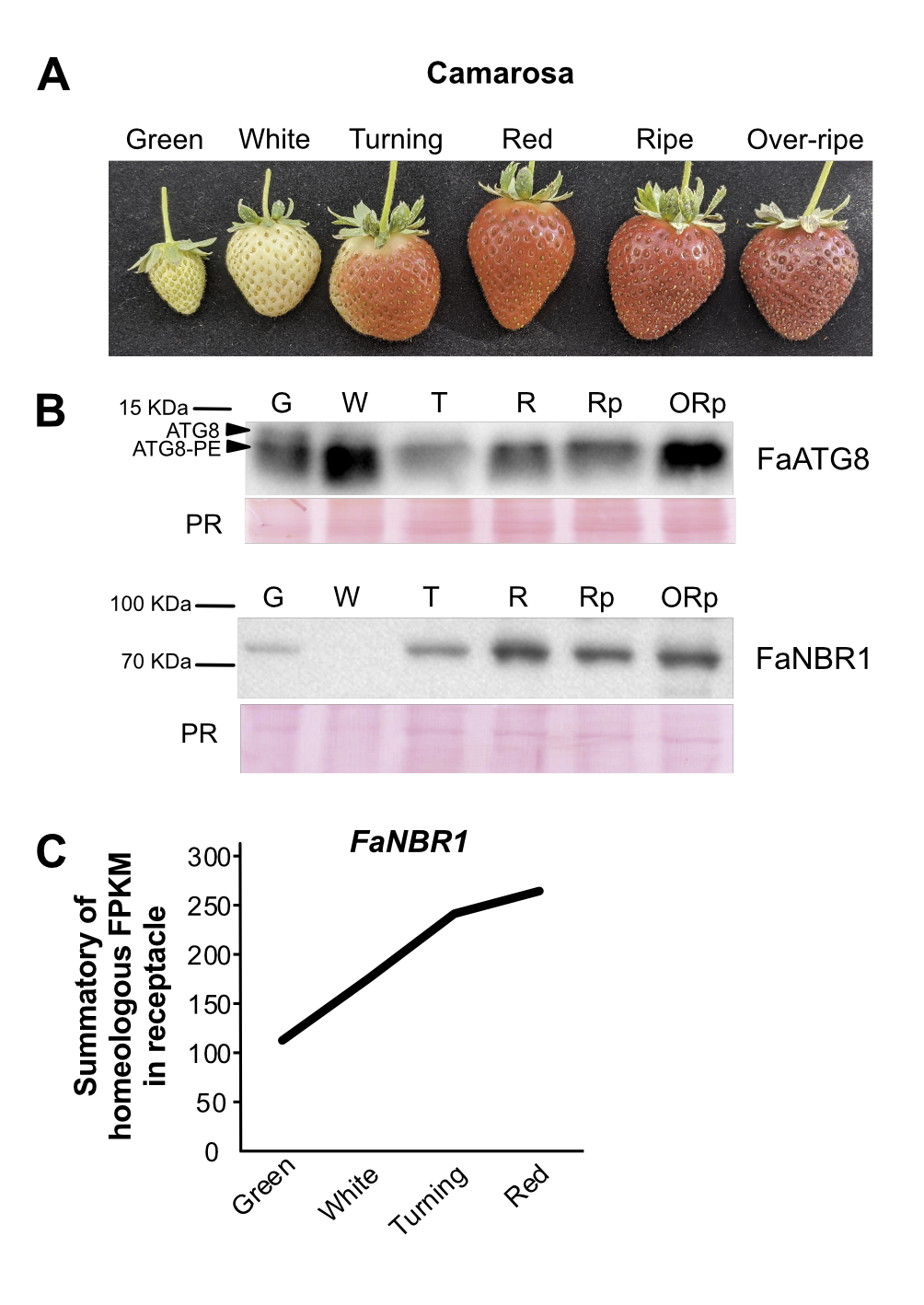

Supplement: Supplementary Figure 2 — Analysis of autophagy flux in F. x ananassa cv. “Camarosa,” (A) Stages of strawberry ripening in F. x ananassa cv. “Camarosa” analyzed for autophagy flux. (B) Immunoblot of ATG8 showing non-lipidated (ATG8) and lipidated form (ATG8-PE) and immunoblot of NBR1 along ripening. (C) Summatory of all NBR1 homoeologs RNAseq data. [file Image_2.TIFF]
