# Supplementary material for: Autophagy Is Required for Strawberry Fruit Ripening
Source: Front Plant Sci. 2021 Aug 27;12:688481. doi: 10.3389/fpls.2021.688481 (PMC8429490; doi:10.3389/fpls.2021.688481)
Supplement: Supplementary Data Sheet 1 — Alignment of the FaATG8 nucleotides sequences that generated phylogenetic tree shown in Figure 2A. [file Data_Sheet_1.docx]

CLUSTAL format alignment by MAFFT (v7.475)

FvH4_3g22920 atg--------------------------g------------------------------

FvH4_3g01980 atg--------------------------g------------------------------

FvH4_6g43130 atgttgggtt--ttagcagaaacgttgtcg------------------------------

FvH4_7g09231 -tgttggattactatccagaaacgtt---g------------------------------

FvH4_7g11130 atg--------------------------a------------------------------

FvH4_5g00060 atg--------------------------g------------------------------

FxaC_12g01440 ATG--------------------------G------------------------------

FxaC_9g01870 ATG--------------------------G------------------------------

FxaC_11g46890 ATG--------------------------G------------------------------

FxaC_12g25380 ATG--------------------------GGCCTCAAGTACATGGTTTGGATCAGCGCCA

FxaC_9g29910 ATG--------------------------T------------------------------

FxaC_11g22890 atg--------------------------g------------------------------

FxaC_12g25610 atg--------------------------g------------------------------

FxaC_10g25330 atg--------------------------g------------------------------

FxaC_12g48710 atg--------------------------g------------------------------

FxaC_10g04960 atg--------------------------g------------------------------

FxaC_23g56560 atgttg-----------------------g------------------------------

FxaC_22g12060 atgttg-----------------------g------------------------------

FxaC_21g14090 atgttg-----------------------g------------------------------

FxaC_26g29480 ------------------------------------------------------------

FxaC_28g28700 -tgttg-----------------------g------------------------------

FxaC_28g26540 at----------------------------------------------------------

FxaC_26g27290 at----------------------------------------------------------

FxaC_26g27370 at----------------------------------------------------------

FxaC_25g27700 at----------------------------------------------------------

FxaC_25g16410 at----------------------------------------------------------

FxaC_27g20200 at----------------------------------------------------------

FxaC_27g13830 at----------------------------------------------------------

FxaC_17g00340 at----------------------------------------------------------

FxaC_18g46510 at----------------------------------------------------------

FxaC_19g00020 at----------------------------------------------------------

FxaC_17g00370 at----------------------------------------------------------

FxaC_25g13980 ------------------------------------------------------------

FvH4_4g21320 at----------------------------------------------------------

FvH4_1g26480 at----------------------------------------------------------

FxaC_1g32800 AT----------------------------------------------------------

FxaC_2g36930 AT----------------------------------------------------------

FxaC_4g08940 AT----------------------------------------------------------

FxaC_13g24640 at----------------------------------------------------------

FxaC_14g17270 at----------------------------------------------------------

FxaC_15g17730 at----------------------------------------------------------

FxaC_27g31780 at----------------------------------------------------------

FvH4_6g19421 at----------------------------------------------------------

FxaC_21g44840 at----------------------------------------------------------

FvH4_3g22920 ------------------------------------------------------------

FvH4_3g01980 ------------------------------------------------------------

FvH4_6g43130 ------------------------------------------------------------

FvH4_7g09231 ------------------------------------------------------------

FvH4_7g11130 ------------------------------------------------------------

FvH4_5g00060 ------------------------------------------------------------

FxaC_12g01440 ------------------------------------------------------------

FxaC_9g01870 ------------------------------------------------------------

FxaC_11g46890 ------------------------------------------------------------

FxaC_12g25380 TTTCTGCTGTATATGCTTGTGTTGCTGCTGTGTCCTCATGGATCAGATGCTTAGTCTCTA

FxaC_9g29910 ------------------------------------------------------------

FxaC_11g22890 ------------------------------------------------------------

FxaC_12g25610 ------------------------------------------------------------

FxaC_10g25330 ------------------------------------------------------------

FxaC_12g48710 ------------------------------------------------------------

FxaC_10g04960 ------------------------------------------------------------

FxaC_23g56560 ------------------------------------------------------------

FxaC_22g12060 ------------------------------------------------------------

FxaC_21g14090 ------------------------------------------------------------

FxaC_26g29480 ------------------------------------------------------------

FxaC_28g28700 ------------------------------------------------------------

FxaC_28g26540 ------------------------------------------------------------

FxaC_26g27290 ------------------------------------------------------------

FxaC_26g27370 ------------------------------------------------------------

FxaC_25g27700 ------------------------------------------------------------

FxaC_25g16410 ------------------------------------------------------------

FxaC_27g20200 ------------------------------------------------------------

FxaC_27g13830 ------------------------------------------------------------

FxaC_17g00340 ------------------------------------------------------------

FxaC_18g46510 ------------------------------------------------------------

FxaC_19g00020 ------------------------------------------------------------

FxaC_17g00370 ------------------------------------------------------------

FxaC_25g13980 ------------------------------------------------------------

FvH4_4g21320 ------------------------------------------------------------

FvH4_1g26480 ------------------------------------------------------------

FxaC_1g32800 ------------------------------------------------------------

FxaC_2g36930 ------------------------------------------------------------

FxaC_4g08940 ------------------------------------------------------------

FxaC_13g24640 ------------------------------------------------------------

FxaC_14g17270 ------------------------------------------------------------

FxaC_15g17730 ------------------------------------------------------------

FxaC_27g31780 ------------------------------------------------------------

FvH4_6g19421 ------------------------------------------------------------

FxaC_21g44840 ------------------------------------------------------------

FvH4_3g22920 ---------------------ccaa-----------------------------------

FvH4_3g01980 ---------------------ccaa-----------------------------------

FvH4_6g43130 ---------------------tcaa-----------------------------------

FvH4_7g09231 ---------------------tcaa-----------------------------------

FvH4_7g11130 ---------------------cgaa-----------------------------------

FvH4_5g00060 ---------------------caaa-----------------------------------

FxaC_12g01440 ---------------------CCAA-----------------------------------

FxaC_9g01870 ---------------------CCAA-----------------------------------

FxaC_11g46890 ---------------------CCAA-----------------------------------

FxaC_12g25380 AAGCTTGGCTTTTCTTTGTCTCTGACCAGATAGTGACCTACTTAATGGTCACATCTGTGG

FxaC_9g29910 ---------------------CGAA-----------------------------------

FxaC_11g22890 ---------------------ccaa-----------------------------------

FxaC_12g25610 ---------------------ccaa-----------------------------------

FxaC_10g25330 ---------------------ccaa-----------------------------------

FxaC_12g48710 ---------------------ccaa-----------------------------------

FxaC_10g04960 ---------------------ctaa-----------------------------------

FxaC_23g56560 ---------------------gttt-----------------------------------

FxaC_22g12060 ---------------------gttt-----------------------------------

FxaC_21g14090 ---------------------gttt-----------------------------------

FxaC_26g29480 ------------------------------------------------------------

FxaC_28g28700 ---------------------gttt-----------------------------------

FxaC_28g26540 ------------------------------------------------------------

FxaC_26g27290 ------------------------------------------------------------

FxaC_26g27370 ------------------------------------------------------------

FxaC_25g27700 ------------------------------------------------------------

FxaC_25g16410 ------------------------------------------------------------

FxaC_27g20200 ------------------------------------------------------------

FxaC_27g13830 ------------------------------------------------------------

FxaC_17g00340 ------------------------------------------------------------

FxaC_18g46510 ------------------------------------------------------------

FxaC_19g00020 ------------------------------------------------------------

FxaC_17g00370 ------------------------------------------------------------

FxaC_25g13980 ------------------------------------------------------------

FvH4_4g21320 ------------------------------------------------------------

FvH4_1g26480 ------------------------------------------------------------

FxaC_1g32800 ------------------------------------------------------------

FxaC_2g36930 ------------------------------------------------------------

FxaC_4g08940 ------------------------------------------------------------

FxaC_13g24640 ------------------------------------------------------------

FxaC_14g17270 ------------------------------------------------------------

FxaC_15g17730 ------------------------------------------------------------

FxaC_27g31780 ------------------------------------------------------------

FvH4_6g19421 ------------------------------------------------------------

FxaC_21g44840 ------------------------------------------------------------

FvH4_3g22920 ------------------------------------------------------------

FvH4_3g01980 ------------------------------------------------------------

FvH4_6g43130 ------------------------------------------------------------

FvH4_7g09231 ------------------------------------------------------------

FvH4_7g11130 ------------------------------------------------------------

FvH4_5g00060 ------------------------------------------------------------

FxaC_12g01440 ------------------------------------------------------------

FxaC_9g01870 ------------------------------------------------------------

FxaC_11g46890 ------------------------------------------------------------

FxaC_12g25380 CTGCAGTGATGGAGATGGTGTTTCTAGCTTATAAAGGTGACAAGGCAGTCTCATGGAGTG

FxaC_9g29910 ------------------------------------------------------------

FxaC_11g22890 ------------------------------------------------------------

FxaC_12g25610 ------------------------------------------------------------

FxaC_10g25330 ------------------------------------------------------------

FxaC_12g48710 ------------------------------------------------------------

FxaC_10g04960 ------------------------------------------------------------

FxaC_23g56560 ------------------------------------------------------------

FxaC_22g12060 ------------------------------------------------------------

FxaC_21g14090 ------------------------------------------------------------

FxaC_26g29480 ------------------------------------------------------------

FxaC_28g28700 ------------------------------------------------------------

FxaC_28g26540 ------------------------------------------------------------

FxaC_26g27290 ------------------------------------------------------------

FxaC_26g27370 ------------------------------------------------------------

FxaC_25g27700 ------------------------------------------------------------

FxaC_25g16410 ------------------------------------------------------------

FxaC_27g20200 ------------------------------------------------------------

FxaC_27g13830 ------------------------------------------------------------

FxaC_17g00340 ------------------------------------------------------------

FxaC_18g46510 ------------------------------------------------------------

FxaC_19g00020 ------------------------------------------------------------

FxaC_17g00370 ------------------------------------------------------------

FxaC_25g13980 ------------------------------------------------------------

FvH4_4g21320 ------------------------------------------------------------

FvH4_1g26480 ------------------------------------------------------------

FxaC_1g32800 ------------------------------------------------------------

FxaC_2g36930 ------------------------------------------------------------

FxaC_4g08940 ------------------------------------------------------------

FxaC_13g24640 ------------------------------------------------------------

FxaC_14g17270 ------------------------------------------------------------

FxaC_15g17730 ------------------------------------------------------------

FxaC_27g31780 ------------------------------------------------------------

FvH4_6g19421 ------------------------------------------------------------

FxaC_21g44840 ------------------------------------------------------------

FvH4_3g22920 ------------------------------------------------------------

FvH4_3g01980 ------------------------------------------------------------

FvH4_6g43130 ------------------------------------------------------------

FvH4_7g09231 ------------------------------------------------------------

FvH4_7g11130 ------------------------------------------------------------

FvH4_5g00060 ------------------------------------------------------------

FxaC_12g01440 ------------------------------------------------------------

FxaC_9g01870 ------------------------------------------------------------

FxaC_11g46890 ------------------------------------------------------------

FxaC_12g25380 AAGCTTGTGCTTCAAATGGGAAATTCTGCAGCAGAATGAAATCGGCCTTGGTTCTCCATT

FxaC_9g29910 ------------------------------------------------------------

FxaC_11g22890 ------------------------------------------------------------

FxaC_12g25610 ------------------------------------------------------------

FxaC_10g25330 ------------------------------------------------------------

FxaC_12g48710 ------------------------------------------------------------

FxaC_10g04960 ------------------------------------------------------------

FxaC_23g56560 ------------------------------------------------------------

FxaC_22g12060 ------------------------------------------------------------

FxaC_21g14090 ------------------------------------------------------------

FxaC_26g29480 ------------------------------------------------------------

FxaC_28g28700 ------------------------------------------------------------

FxaC_28g26540 ------------------------------------------------------------

FxaC_26g27290 ------------------------------------------------------------

FxaC_26g27370 ------------------------------------------------------------

FxaC_25g27700 ------------------------------------------------------------

FxaC_25g16410 ------------------------------------------------------------

FxaC_27g20200 ------------------------------------------------------------

FxaC_27g13830 ------------------------------------------------------------

FxaC_17g00340 ------------------------------------------------------------

FxaC_18g46510 ------------------------------------------------------------

FxaC_19g00020 ------------------------------------------------------------

FxaC_17g00370 ------------------------------------------------------------

FxaC_25g13980 ------------------------------------------------------------

FvH4_4g21320 ------------------------------------------------------------

FvH4_1g26480 ------------------------------------------------------------

FxaC_1g32800 ------------------------------------------------------------

FxaC_2g36930 ------------------------------------------------------------

FxaC_4g08940 ------------------------------------------------------------

FxaC_13g24640 ------------------------------------------------------------

FxaC_14g17270 ------------------------------------------------------------

FxaC_15g17730 ------------------------------------------------------------

FxaC_27g31780 ------------------------------------------------------------

FvH4_6g19421 ------------------------------------------------------------

FxaC_21g44840 ------------------------------------------------------------

FvH4_3g22920 -------------------------------------------------aagc-tcct--

FvH4_3g01980 -------------------------------------------tgacaaaagc-ttgt--

FvH4_6g43130 -------------------------------------------------gaag-tctt--

FvH4_7g09231 -------------------------------------------------caag-tctt--

FvH4_7g11130 -------------------------------------------------aagt-acct--

FvH4_5g00060 -------------------------------------------------gagt-tact--

FxaC_12g01440 -------------------------------------------TAACAAAAGT-TTGT--

FxaC_9g01870 -------------------------------------------TAACAAAAGT-TTGT--

FxaC_11g46890 -------------------------------------------TAACAAAAGT-TTGT--

FxaC_12g25380 CTCTTGCCCTTTTTAGCTTCCTTGTTTTAGCTGTGATTTCTGCTTATAGGATC-TTCA--

FxaC_9g29910 -------------------------------------------------AA------T--

FxaC_11g22890 -------------------------------------------------aagc-tcct--

FxaC_12g25610 -------------------------------------------------aagc-tcct--

FxaC_10g25330 -------------------------------------------------aagc-tcct--

FxaC_12g48710 -------------------------------------------tgacaaaagc-ttgt--

FxaC_10g04960 -------------------------------------------tgacaaaagc-ttgt--

FxaC_23g56560 -------------------------------------------tagcagaaacgttgtcg

FxaC_22g12060 -------------------------------------------tagcagaaacgttgtcg

FxaC_21g14090 -------------------------------------------tagcagaaacgttgtcg

FxaC_26g29480 ------------------------------------------------------------

FxaC_28g28700 -------------------------------------------tagcagaaacatt---g

FxaC_28g26540 -----------------------------------------------------------g

FxaC_26g27290 -----------------------------------------------------------g

FxaC_26g27370 -----------------------------------------------------------g

FxaC_25g27700 -----------------------------------------------------------g

FxaC_25g16410 -----------------------------------------------------------g

FxaC_27g20200 -----------------------------------------------------------g

FxaC_27g13830 -----------------------------------------------------------g

FxaC_17g00340 -----------------------------------------------------------g

FxaC_18g46510 -----------------------------------------------------------g

FxaC_19g00020 -----------------------------------------------------------g

FxaC_17g00370 -----------------------------------------------------------g

FxaC_25g13980 ------------------------------------------------------------

FvH4_4g21320 -----------------------------------------------------------g

FvH4_1g26480 -----------------------------------------------------------g

FxaC_1g32800 -----------------------------------------------------------G

FxaC_2g36930 -----------------------------------------------------------G

FxaC_4g08940 -----------------------------------------------------------G

FxaC_13g24640 -----------------------------------------------------------g

FxaC_14g17270 -----------------------------------------------------------g

FxaC_15g17730 -----------------------------------------------------------g

FxaC_27g31780 -----------------------------------------------------------g

FvH4_6g19421 -----------------------------------------------------------g

FxaC_21g44840 -----------------------------------------------------------g

FvH4_3g22920 tcaagct-------------agagcacc-------ctct-----------------cgag

FvH4_3g01980 tcaagaa-------------acaacatg-------ctct-----------------tgag

FvH4_6g43130 acaagtc-------------ggaagtcc-------ctct-----------------cgag

FvH4_7g09231 acaagtc-------------ggaagtcc-------cttt-----------------cgag

FvH4_7g11130 tcaagca-------------agcacatg-------agtt-----------------tgag

FvH4_5g00060 tcaagca-------------agaacatg-------atct-----------------tgag

FxaC_12g01440 TCAAGAA-------------AAAACATC-------CTCT-----------------TGAG

FxaC_9g01870 TCAAGAA-------------AAAACATC-------CTCT-----------------TGAG

FxaC_11g46890 TCAAGAA-------------AAAACATC-------CTCT-----------------TGAG

FxaC_12g25380 GCATCTG----T--------GAGGCACC-------TTCTGTTTCTCACAAAGAGTGGAAG

FxaC_9g29910 TCAAAAT-------------TTGACATG-------T------------GACAAGACAAAG

FxaC_11g22890 tcaagct-------------agaacacc-------ctct-----------------cgag

FxaC_12g25610 tcaagct-------------agaacacc-------ctct-----------------cgag

FxaC_10g25330 tcaagct-------------ggaacacc-------ctct-----------------cgag

FxaC_12g48710 tcaagaa-------------acaacatg-------ctct-----------------tgag

FxaC_10g04960 ttaagaa-------------acaacatg-------ctct-----------------tgag

FxaC_23g56560 tcaagaa----gt----cttacaagtcggatgtccctct-----------------cgag

FxaC_22g12060 tcaagaa----gt----cttacaagtcggaagtccctct-----------------cgag

FxaC_21g14090 tcaagaa----gt----cttacaagtcggaagtccctct-----------------cgag

FxaC_26g29480 ------------------------------------------------------------

FxaC_28g28700 tcaacaa----gt----cttacaagtcggaagtcccttt-----------------cgag

FxaC_28g26540 acgaaaa----gt---accttcaagcaagcacatgagtt-----------------tgag

FxaC_26g27290 acgaaaa----gt---accttcaagcaagcacatgagtt-----------------tgag

FxaC_26g27370 acgaaaa----gt---accttcaagcaagcacatgagtt-----------------tgag

FxaC_25g27700 acgaaaa----gt---accttcaagcaagcacatgagtt-----------------tgag

FxaC_25g16410 acgaaaa----gt---accttcaagcaagcacatgagtt-----------------tgag

FxaC_27g20200 acgaaaa----gt---accttcaagcaagcacatgagtt-----------------tgag

FxaC_27g13830 acgaaaa----gt---accttcaagcaagcacatgagtt-----------------tgag

FxaC_17g00340 -----------------tgtttgtgt-----------tc-----------------agag

FxaC_18g46510 gcaaaga----gt---tacttcaagcaagaacatgatct-----------------tgag

FxaC_19g00020 gcaaaga----gt---tacttcaagcaagaacatgatct-----------------tgag

FxaC_17g00370 gcaaaga----gt---tacttcaagcaagaacatgatct-----------------tgag

FxaC_25g13980 ------------------------------------------------------------

FvH4_4g21320 ggcaaga----tcacgtctttcaagcaagagttctcttt-----------------cgat

FvH4_1g26480 ggtagaa----caaagtcattcaaggatgagcttacgtt-----------------cgaa

FxaC_1g32800 GGTAGAA----CAAAGTCATTCAAGGATGAGCTTACGTT-----------------CGAA

FxaC_2g36930 GGTAGAA----CGAAGTCATTCAAGGATGAGCTTACGTT-----------------CGAA

FxaC_4g08940 GGTAGAA----CGAAGTCATTCAAGGATGAGCTTACGTT-----------------CGAA

FxaC_13g24640 ggcaaga----tcacgtctttcaagcaagagttctcttt-----------------cgat

FxaC_14g17270 ggcaaga----tcacgtctttcaagcaagagttctcttt-----------------cgac

FxaC_15g17730 ggcaaga----tcacgtctttcaagcaagagttctcttt-----------------cgat

FxaC_27g31780 ggtagaa----caaagtcattcaaggatgagcttacgtt-----------------cgaa

FvH4_6g19421 tcgaagatactgagaatcactcccggtgaggattgttcc---------------------

FxaC_21g44840 tcgaagatactgagaatcactcccggtgaggattgttcc---------------------

FvH4_3g22920 aggaggcaggcagaagctgctcgtatcagggagaag--tatcctgacc-----gaattcc

FvH4_3g01980 aggaggaaggcagaagctttgcgaatcagggagaag--tatccggaga-----gagtacc

FvH4_6g43130 aaaagggaagctgaagctgctcgcatcagagagcag--catcctgata-----gagtacc

FvH4_7g09231 aaaagggaagcagaagctgctcgcattagagagcat--catcctgata-----gagtacc

FvH4_7g11130 aagaggcgtgctgaggcctcgaggatcagagagaag--tacccagata-----gaattcc

FvH4_5g00060 aagaggagggctgaggctgctaggattagggagaaa--tacccggata-----ggattcc

FxaC_12g01440 AGGAGGAAGGCAGAAGCTTTGCGAATCAGGGAGAAG--CACCCGGAGA-----AAGTACC

FxaC_9g01870 AGGAGGAAGGCAGAAGCTTTGCGAATCAGGGGGAAG--CATCCGGAGA-----AAGTACC

FxaC_11g46890 AGGAGGAAGGCAGAAGCTTTGCGAATCAGGGAGAAG--CATCCGGAGA-----AAGTACC

FxaC_12g25380 AGGAGGCAGGCAGAAGCTGCTCGTATCAGGGAGAAG--TATCCTGACC-----GAATTCC

FxaC_9g29910 AGGAGGCAGGCAGAAGCTGCTCGTATCAGGGAGAAG--TATCCTGACC-----GAATTCC

FxaC_11g22890 aggaggcaggcagaagctgctcgtatcagggagaag--tatcctgacc-----gaattcc

FxaC_12g25610 aggaggcaggcagaagctgctcgtatcagggagaag--tatcctgacc-----gaattcc

FxaC_10g25330 aggaggcaggcagaagctgctcgtatcagggagaaa--tatcctgacc-----gaattcc

FxaC_12g48710 aggaggaaggcagaagctgtgcgaatcagggagaag--tatccggaga-----gagtacc

FxaC_10g04960 aggaggaagtcagaagctgtgagaatcagggagaag--tatccggaga-----gagtacc

FxaC_23g56560 aaaagggaagccgaagctgctcgcatcagagagcag--catcctgata-----gagtacc

FxaC_22g12060 aaaagggaagccgaagctgctcgcatcagagagcaa--catcctgata-----gagtacc

FxaC_21g14090 aaaagggaagctgaagctgctcgcatcagagagcag--catcctgata-----gagtacc

FxaC_26g29480 ----------ccgaagctgcccgcattagagagcat--catcctgata-----gagtacc

FxaC_28g28700 aaaagggaagcagaagctgctcgcattagagagcat--catcctgata-----gagtacc

FxaC_28g26540 aagaggcgtgctgaggcctcgaggatcagagagaag--tacccagata-----gaattcc

FxaC_26g27290 aagaggcgtgctgaggcctcgaggatcagagagaag--tacccagata-----gaattcc

FxaC_26g27370 aagaggcgtgctgaggcctcgaggatcagagagaag--tacccagata-----gaattcc

FxaC_25g27700 aagaggcgtgctgaggcctcgaggatcagagagaag--tacccagata-----gaattcc

FxaC_25g16410 aagaggcgtgctgaggcctcgaggatcagagagaag--tacccagata-----gaattcc

FxaC_27g20200 aagaggcgtgctgaggcctcgaggatcagagagaag--tacccagata-----gaattcc

FxaC_27g13830 aagaggcgtgctgaggcctcgaggatcagagagaag--taccctgata-----gaattcc

FxaC_17g00340 aagaggagggctgaggctgctaggattagggagaaa--tacccggata-----ggattcc

FxaC_18g46510 aggaggagggctgaggctgctaggattagggagaaa--tacccagata-----ggattcc

FxaC_19g00020 aagaggagggctgaggctgctaggattagggagaaa--tacccggata-----ggattcc

FxaC_17g00370 aagaggagggctgaggctgctaggattagggagaaa--tacccggata-----ggattcc

FxaC_25g13980 ------gaagcagaagctgctcgcattagagagcat--catcctgata-----gagtacc

FvH4_4g21320 gagcgtcttgaagaatcaaagaacatcattggaaag--taccctgatc-----gagttcc

FvH4_1g26480 caaagggtggaagaatcgcggaatattctggccaat--taccctgatc-----gagttcc

FxaC_1g32800 CAAAGGGTGGAAGAATCGCGGAATATTCTGGCCAAT--TACCCTGATC-----GAGTTCC

FxaC_2g36930 CAGAGGGTGGAAGAATCGCGGAATATTCTGGCCAAT--TACCCTGATC-----GCGTTCC

FxaC_4g08940 CAGAGGGTGGAAGAATCGCGGAATATTCTGGCCAAT--TACCCTGATC-----GCGTTCC

FxaC_13g24640 gagcgtcttgaagaatcaaagaacatcattggaaag--taccctgatc-----gagttcc

FxaC_14g17270 gagcgtcttgaagaatcaaagaacatcattggaaag--tacccagatc-----gagttcc

FxaC_15g17730 gagcgtcttgaggaatcaaagaacatcattggaaag--tacccagatc-----gagttcc

FxaC_27g31780 caaagggtggaagaatcgcggaatattctggccaat--taccctgatc-----gagttcc

FvH4_6g19421 ------agtgaaacttccacccatgtcatcggcaatggcagccatatcagggggaacttc

FxaC_21g44840 ------agtgaaacttccacccatgtcatcggcaatggcagccatatcagggggaacttc

. * . .*. * * .* ** * . .. .*

FvH4_3g22920 g---gttattgtg-------gagaaggct-gaaaggagtgacataccagacattgacaag

FvH4_3g01980 g---gtgattgtg-------gagaaggcc-gtaaagagtgacgttcctgacattgacaag

FvH4_6g43130 g---gtgattgtt-------gaaaaggct-gaaaagagtgatgtgcctgatattgagaag

FvH4_7g09231 a---gtgattgtt-------gaaaaggcg-aacaaaagtgatgtgcctgatattgagaag

FvH4_7g11130 a---gtgattgtg-------gaaaaggca-gagagaagtgatattcccaacattgacaag

FvH4_5g00060 g---gtgattgtg-------gagaaggca-gaaagaagtgatatcccaaacattgataag

FxaC_12g01440 A---GTGATTGTG-------GAGAAGGCC-ATAAAGAGTGACATTGCTGACATTGACAAG

FxaC_9g01870 A---GTGATTGTG-------GAGAAGGCC-ATAAAGAGTGACATTGCTGACATTGACAAG

FxaC_11g46890 A---GTGATTGTG-------GAGAAGGCC-ATAAAGAGTGACATTGCTGACATTGACAAG

FxaC_12g25380 G---GTTATTGTG-------GAGAAGGCT-GAAAGGAGTGACATACCAGACATTGACAAG

FxaC_9g29910 G---GTTATTGTG-------GAGAAGGCT-GAAAGGAGTGACATACCAGACATTGACAAG

FxaC_11g22890 g---gttatcgtg-------gagaaggct-gaaaggagtgacataccagacattgacaag

FxaC_12g25610 g---gttattgtg-------gagaaggct-gaaaggagtgacataccagacattgacaag

FxaC_10g25330 g---gttattgtg-------gagaaggct-gaaaggagtgacataccagacattgacaag

FxaC_12g48710 g---gcaa-------------------ct-tcaagaactaata------acattcacaag

FxaC_10g04960 a---gtgattgtg-------gagaaggcc-ggaaagagtgacgttcctgacattgacaag

FxaC_23g56560 g---gtgattgtt-------gaaaaggct-gaaaagagtgatgtgcctgatattgagaag

FxaC_22g12060 g---gtgattgtt-------gaaaaggct-gaaaagagtgatgtgcctgatattgagaag

FxaC_21g14090 g---gtgattgtt-------gaaaaggct-gaaaagagtgatgtgcctgatattgagaag

FxaC_26g29480 g---gtgattgtt-------gaaaaggct-aacaaaagtgatgtgcctgatattgagaaa

FxaC_28g28700 a---gtgattgtt-------gaaaaggct-aacaaaagtgatgtgcctgatattgagaag

FxaC_28g26540 a---gtgattgtg-------gagaaggca-gagagaagtgatattcccaacattgacaag

FxaC_26g27290 a---gtgattgtg-------gagaaggca-gagagaagtgatattcccaacattgacaag

FxaC_26g27370 a---gtgattgtg-------gagaaggca-gagagaagtgatattcccaacattgacaag

FxaC_25g27700 a---gtgattgtg-------gaaaaggca-gagagaagtgatattcccaacattgacaag

FxaC_25g16410 a---gtgattgtg-------gaaaaggca-gagagaagtgatattcccaacattgacaag

FxaC_27g20200 a---gtgattgtg-------gaaaaggca-gagagaagtgatattcccaacattgacaag

FxaC_27g13830 a---gtgattgtg-------gaaaaggca-gagagaagtgatattcccaacattgacaag

FxaC_17g00340 g---gtgattgtg-------gagaaggca-gaaagaagtgatatcccaaacattgataag

FxaC_18g46510 g---gtgattgtg-------gagaaggca-gaaagaagtgatatcccaaacattgataag

FxaC_19g00020 g---gtgattgtg-------gagaaggca-gaaagaagtgatatcccaaacattgataag

FxaC_17g00370 g---gtgattgtg-------gagaaggca-gaaagaagtgatatcccaaacattgataag

FxaC_25g13980 a---gtgattgtt-------gaaaaggcg-aacaaaagtgat------------------

FvH4_4g21320 g---gttattatt-------gaaagatat-tcgaggacagacctgcctgaaatggaaaag

FvH4_1g26480 g---gtgattgtt-------gaaaagtat-gccaagtgcgatcttcctcagctggataag

FxaC_1g32800 G---GTGATTGTT-------GAAAAGTAT-GCCAAGTGCGATCTTCCTCAGCTGGATAAG

FxaC_2g36930 G---GTGATTGTT-------GAAAAGTAT-GCCAAGTGCGATCTTCCTCAGCTGGATAAG

FxaC_4g08940 G---GTGATTGTT-------GAAAAGTAT-GCCAAGTGCGATCTTCCTCAGCTGGATAAG

FxaC_13g24640 g---gttattatt-------gaaagatat-tcgaggacagacctgcctgaaatggaaaag

FxaC_14g17270 g---gttattatt-------gaaagatat-tcgaggacagacctgcctgaaatggaaaag

FxaC_15g17730 g---gttattatt-------gaaagatat-tcgaggacagacctgcctgaaatggaaaag

FxaC_27g31780 g---gtgattgtt-------gaaaagtat-gccaagtgcgatcttcctcagctggataag

FvH4_6g19421 aaacgcaatcattctctggagaaaaggcgagcccaaatagagcg---taacaag------

FxaC_21g44840 aaacgcaatcattctctggagaaaaggcgaacccaaatagagcgtgttagcagggagata

. *. * .. .*

FvH4_3g22920 ---aagaag---------------------------------------------------

FvH4_3g01980 ---aagaaa---------------------------------------------------

FvH4_6g43130 ---aacaaa---------------------------------------------------

FvH4_7g09231 ---aacaaa---------------------------------------------------

FvH4_7g11130 ---aaaaaa---------------------------------------------------

FvH4_5g00060 ---aaaaag---------------------------------------------------

FxaC_12g01440 ---AACAAG---------------------------------------------------

FxaC_9g01870 ---AACAAG---------------------------------------------------

FxaC_11g46890 ---AACAAG---------------------------------------------------

FxaC_12g25380 ---AAGAAG---------------------------------------------------

FxaC_9g29910 ---AAGAAG---------------------------------------------------

FxaC_11g22890 ---aagaag---------------------------------------------------

FxaC_12g25610 ---aagaag---------------------------------------------------

FxaC_10g25330 ---aagaag---------------------------------------------------

FxaC_12g48710 atcaaggga---------------------------------------------------

FxaC_10g04960 ---aagaaa---------------------------------------------------

FxaC_23g56560 ---aacaaa---------------------------------------------------

FxaC_22g12060 ---aacaaa---------------------------------------------------

FxaC_21g14090 ---aacaaa---------------------------------------------------

FxaC_26g29480 ---aacaaa---------------------------------------------------

FxaC_28g28700 ---aacaaa---------------------------------------------------

FxaC_28g26540 ---aaaaaa---------------------------------------------------

FxaC_26g27290 ---aaaaaa---------------------------------------------------

FxaC_26g27370 ---aaaaaa---------------------------------------------------

FxaC_25g27700 ---aaaaaa---------------------------------------------------

FxaC_25g16410 ---aaaaaa---------------------------------------------------

FxaC_27g20200 ---aaaaaa---------------------------------------------------

FxaC_27g13830 ---aaaaaa---------------------------------------------------

FxaC_17g00340 ---aaaaag---------------------------------------------------

FxaC_18g46510 ---aaaaag---------------------------------------------------

FxaC_19g00020 ---aaaaag---------------------------------------------------

FxaC_17g00370 ---aaaaag---------------------------------------------------

FxaC_25g13980 ------------------------------------------------------------

FvH4_4g21320 ---aaaaaa---------------------------------------------------

FvH4_1g26480 ---agaaaa---------------------------------------------------

FxaC_1g32800 ---AGAAAA---------------------------------------------------

FxaC_2g36930 ---AGAAAA---------------------------------------------------

FxaC_4g08940 ---AGAAAA---------------------------------------------------

FxaC_13g24640 ---aaaaaa---------------------------------------------------

FxaC_14g17270 ---aaaaaa---------------------------------------------------

FxaC_15g17730 ---aaaaaa---------------------------------------------------

FxaC_27g31780 ---agaaaa---------------------------------------------------

FvH4_6g19421 ------------------------------------------------------------

FxaC_21g44840 tatccagaaagaatacctgtgattaattgtggagagggctatattcaccgacgttgcttg

FvH4_3g22920 ---tatctggttcctgctgatctgactgttgggcagttcgtttatgtggtccggaaaagg

FvH4_3g01980 ---taccttgtgcctgctgatttgactgttggacaatttggttatgttgtccgcaagagg

FvH4_6g43130 ---tacctggtgcctgccgatattactgttgggcagttagtctttgtgatccggaaaaag

FvH4_7g09231 ---tacctggtgcctgccgatattactgttgggcatttagtctttgtgatccggaaaaag

FvH4_7g11130 ---tacctagtaccagctgatttgactgttggtcaatttgtctatgtcatccgcaagagg

FvH4_5g00060 ---taccttgttccggccgatctaactgtaggacaatttgtttatgtcatccgcaaaagg

FxaC_12g01440 ---TACCTTGTGCCTGGTGATTTCACTGTTGGACACTTTGCTTATGTTGTCCGCGAGAGG

FxaC_9g01870 ---TACCTTGTGCCTGGTGATTTCACTGTTGGACACTTTGCTTATGTTGTCCGCGAGAGG

FxaC_11g46890 ---TACCTTGTGCCTGGTGATTTCACTGTTGGACACTTTGCTTATGTTGTCCGCGGGAGG

FxaC_12g25380 ---TATCTGGTTCCTGCTGATCTGACTGTTGGGCAGTTCGTTTATGTGGTCCGGAAGAGG

FxaC_9g29910 ---TATCTGGTTCCTGCTGATCTGACTGTTGGGCAGTTCGTTTATGTGGTCCGGAAAAGG

FxaC_11g22890 ---tatctggttcctgctgatctgactgttgggcagttcgtttatgtggtccggaagagg

FxaC_12g25610 ---tatctggttcctgctgatctgactgttgggcagttcgtttatgtggtccggaagagg

FxaC_10g25330 ---tatctggttcctgctgatctgactgttgggcagttcgtttatgtggtccggaagagg

FxaC_12g48710 ---tactttgtgcctgctgatttgactgttggacaatttggttatgttgtccgcaagagg

FxaC_10g04960 ---taccttgtgcctgctgatttgactgttggacaatttggttatgttgtccgcaagagg

FxaC_23g56560 ---tacctggtgcctgccgatattactgttgggcagttagtctttgtgatccggaaaaag

FxaC_22g12060 ---tacctggtgcctgccgatattactgttgggcagttagtctttgtgatccggaaaaag

FxaC_21g14090 ---tacctggtgcctgccgatattactgttgggcagttagtctttgtgatccggaaaaag

FxaC_26g29480 ---tacctggtgcctgccgatattactgttgggcatttagtctttgtgatccggaaaaag

FxaC_28g28700 ---tacctggtgcctgccgatattactgttgggcatttagtctttgtgatccggaaaaag

FxaC_28g26540 ---tacctagtaccagctgatttgactgttggtcaatttgtctatgtcatccgcaagagg

FxaC_26g27290 ---tacctagtaccagctgatttgactgttggtcaatttgtctatgtcatccgcaagagg

FxaC_26g27370 ---tacctagtaccagctgatttgactgttggtcaatttgtctatgtcatccgcaagagg

FxaC_25g27700 ---tacctagtaccagctgatttgactgttggtcaatttgtctatgtcatccgcaagagg

FxaC_25g16410 ---tacctagtaccagctgatttgactgttggtcaatttgtctatgtcatccgcaagagg

FxaC_27g20200 ---tacctagtaccagctgatttgactgttggtcaatttgtctatgtcatccgcaagagg

FxaC_27g13830 ---tacctagtaccagctgatttgactgttggtcaatttgtctatgtcatccgcaagagg

FxaC_17g00340 ---taccttgttccggccgatctaactgtaggacaatttgtttatgtcatccgcaaaagg

FxaC_18g46510 ---taccttgttccggccgatctaactgtaggacaatttgtttatgtcatccgcaaaagg

FxaC_19g00020 ---taccttgttccggccgatctaactgtaggacaatttgtttatgtcatccgcaaaagg

FxaC_17g00370 ---taccttgttccggccgatctaactgtaggacaatttgtttatgtcatccgcaaaagg

FxaC_25g13980 ------------------------------------------------------------

FvH4_4g21320 ---tacttggttcctagagatatgtctgttggccagtttatccatattttaagcagccgg

FvH4_1g26480 ---tatcttgtcccccgggacacgtctgtcgggcaattcatttacattttgagcgacaga

FxaC_1g32800 ---TATCTTGTCCCCCGGGACATGTCTGTCGGGCAATTCATTTACATTTTGAGCGACAGA

FxaC_2g36930 ---TATCTTGTCCCCCGGGACATGTCTGTCGGGCAATTCATTTACATTTTGAGCGACAGA

FxaC_4g08940 ---TATCTTGTCCCCCGGGACATGTCTGTCGGGCAATTCATTTACATTTTGAGCGACAGA

FxaC_13g24640 ---tacttggttcctagagatatgtctgttggccagtttatccatattttaagcagccgg

FxaC_14g17270 ---tacttggttcctagagatatgtctgttggccagtttatccatattttaagcagccgg

FxaC_15g17730 ---tacttggttcctagagatatgtctgttggccagtttatccatattttaagcagccgg

FxaC_27g31780 ---tatcttgtcccccgggacatgtctgtcgggcaattcatttacattttgagtgacaga

FvH4_6g19421 ---ttccttgtccctgccgatcttactcttggacaatttggttatgctattcgtaagatg

FxaC_21g44840 cattaccttgtccctgccgatcttactcttggacaatttggttatgctattcgtaagatg

FvH4_3g22920 ataaagcttagtgctgagaaggccatctttatatttgtcaagaacattttgccacctact

FvH4_3g01980 atcaagctcggtgcagagaaggctatatttacctttgtcaacaacgttttacctccccaa

FvH4_6g43130 atcaagcttgatgctgaccaggcaattttcgttttcgttaaggatattttgccacctaca

FvH4_7g09231 atcaagcttgatcctgaccaggcaatttttgttttcgttaaggatattttgccaccaaca

FvH4_7g11130 atcaagctgagtgcagaaaaggccatcttcatatttgtggacaacgtcctcccacctaca

FvH4_5g00060 attaaattaagtgcagaaaaggcaatctttatatttgtggacaatgttcttccaccaaca

FxaC_12g01440 ATCAAGCTCGGTGCAGAGAAGGCTATATTTATCTTTGTCAACAACCTTTTACCTCCCCAA

FxaC_9g01870 ATCAAGCTCGGTGCAGAGAAGGCTATATTTATCTTTGTCAACAACCTTTTACCTCCCCAA

FxaC_11g46890 ATCAAGCTCGGTGCAGAGAAGGCTATCTTTATCTTTGTCAACAACCTTTTACCTCCCCAA

FxaC_12g25380 ATAAAGCTTAGTGCTGAGAAGGCCATCTTTATATTTGTCAAGAACATTTTGCCACCTACT

FxaC_9g29910 ATAAAGCTTAGTGCTGAGAAGGCCATCTTTATATTTGTCAAGAACATTTTGCCACCTACT

FxaC_11g22890 ataaagcttagtgctgagaaggccatctttatatttgtcaagaacattttgccacctact

FxaC_12g25610 ataaagcttagtgctgagaaggccatctttatatttgtcaagaacattttgccacctact

FxaC_10g25330 ataaagcttagtgctgagaaggccatctttatatttgtcaagaacattttgccacctact

FxaC_12g48710 atcaagctcggtgcagagaaggctatatttatctttgtcaagaacgttttacctccccaa

FxaC_10g04960 atcaagctcggtgcagagaaggctatatttatctttgtcaagaacattttacctccccaa

FxaC_23g56560 atcaagcttgatgctgaccaggcaattttcgttttcgtcaaggatattttgccacctaca

FxaC_22g12060 atcaagcttgatgctgaccaggcaattttcattttcgtcaaggatattttgccacctaca

FxaC_21g14090 atcaagcttgatgctgaccaggcaattttcgttttcgttaaggatattttgccacctaca

FxaC_26g29480 atcaagcttgatcctgaccaggcaatttttgttttcgttaaggatattttgccaccgaca

FxaC_28g28700 atcaagcttgattctgaccaggcaatttttgttttcgttaaggatattttgccaccaaca

FxaC_28g26540 atcaagctgagtgcagaaaaggccatcttcatatttgtggacaacgtcctcccacctaca

FxaC_26g27290 atcaagctgagtgcagaaaaggccatcttcatatttgtggacaacgtcctcccacctaca

FxaC_26g27370 atcaagctgagtgcagaaaaggccatcttcatatttgtggacaacgtcctcccacctaca

FxaC_25g27700 atcaagctgagtgcagaaaaggccatcttcatatttgtggacaatgtcctcccacctaca

FxaC_25g16410 atcaagctgagtgcagaaaaggccatcttcatatttgtggacaacgtcctcccacctaca

FxaC_27g20200 atcaagctgagtgcagaaaaggccatcttcatatttgtggacaacgtcctcccacctaca

FxaC_27g13830 atcaagctgagtgcagaaaaggccatcttcatatttgtggacaacgtcctcccacctaca

FxaC_17g00340 attaaattaagtgcagaaaaggcaatctttatatttgtggacaatgttcttccaccaaca

FxaC_18g46510 attaaattaagtgcagaaaaggcaatctttatatttgtggacaatgttcttccaccaaca

FxaC_19g00020 attaaattaagtgcagaaaaggcaatctttatatttgtggacaatgttcttccaccaaca

FxaC_17g00370 attaaattaagtgcagaaaaggcaatctttatatttgtggacaatgttcttccaccaaca

FxaC_25g13980 ------------------------------------------gatattttgccaccaaca

FvH4_4g21320 cttcaactgactcctgggaaagcgctttttgtatttgtgaagaacacattacctcaaaca

FvH4_1g26480 cttcgtctacaccctggagaagctctcttcgtattagtacagaatacactacccccaaca

FxaC_1g32800 CTTCGTCTACACCCTGGAGAAGCTCTCTTCGTATTAGTACAGAATACACTACCCCCAACA

FxaC_2g36930 CTTCGTCTACACCCTGGAAAAGCTCTCTTCGTATTTGTACAGAGTACTTTACCCCCAATA

FxaC_4g08940 CTTCGTCTACACCCTGGAAAAGCTCTCTTCGTATTTGTACAGAGTACTTTACCCCCAATA

FxaC_13g24640 cttcaactgactcctgggaaagcgctttttgtatttgtgaagaacacattacctcaaaca

FxaC_14g17270 cttcaactgactcctgggaaagcactttttgtatttgtgaagaacacattacctcaaaca

FxaC_15g17730 cttcaactgactcctgggaaagcactttttgtatttgtgaagaacacattacctcaaaca

FxaC_27g31780 ctttgtctacaccctggaaaagctctcttcgtatttgt-------actttacccccaata

FvH4_6g19421 atccacctcggagcaggcaaggctatatttctcttcgtcaacaacactttacctcgcact

FxaC_21g44840 atccacctcggagcaggcaaggctatatttctcttcgtcaaaaacactttacctcgcact

. .* ** *

FvH4_3g22920 gctgccatgatgtcagcgatctatgaggaaaacaaagacgaggatggtttcctttatat-

FvH4_3g01980 gctgccttgatgtcagcaatttacgaagacaacaaggatgaagatggattcctttacat-

FvH4_6g43130 gctgctttgatgtcttcaatttatgaggaaaataaggacgaggatggttttctttatat-

FvH4_7g09231 gctgctttaatgtcttcaatttatgagaaacgtaaggatgaagatggttttctttatat-

FvH4_7g11130 ggagctgtaatgtctactatctatgatgacaagaaggacggtgatggatttctttacgt-

FvH4_5g00060 ggtgcaattatgtctgccatatatgaagagaagaaggatgaagatggctttctctacgt-

FxaC_12g01440 GCTGCCTTGATGTCTTCAATTTATGAACAAAACAAGGATGAAGATGGATTCCTTTACAT-

FxaC_9g01870 GCTGCCTTGATGTCTTCAATTTATGAACAAAACAAGGATGAAGATGGATTCCTTTACAT-

FxaC_11g46890 GCTGCCTTGATGTCTTCAATTTATGAACAAAACAAGGATGAAGATGGATTCCTGTACAT-

FxaC_12g25380 GCTGCCATGATGTCAGCGATCTATGAGGAAAACAAAGACGAGGATGGTTTCCTTTATAT-

FxaC_9g29910 GCTGCCATGATGTCAGCGATCTATGAGGAAAACAAAGACGAGGATGGTTTCCTTTATAT-

FxaC_11g22890 gctgccatgatgtcagcgatctatgaggaaaacaaagacgaggatggtttcctttatat-

FxaC_12g25610 gctgccatgatgtcagcgatctatgaggaaaacaaagacgaggatggtttcctttatat-

FxaC_10g25330 gctgccatgatgtcagcgatctatgaggaaaacaaagacgaggatggtttcctttatat-

FxaC_12g48710 gctgccttgatgtcagcaatttacaaagacaacaaggatgaagatggattcctttacat-

FxaC_10g04960 gctgccttgatgtcagcaatttacgaagacaacaaggatgaagatggattcctttacat-

FxaC_23g56560 gctgctttgatgtcttcaatttatgaagaaaataaggacggggatggttttctttatat-

FxaC_22g12060 gctgctttgatgtcttcaatttatgaggaaaataaggacgaggatggttttctttatat-

FxaC_21g14090 gctgctttgatgtcttcaatttatgaggaaaataaggacgaggatggttttctttatat-

FxaC_26g29480 gctgctttaacgtcttcaatttatgagaaacataaggatgaagatggttttctttatat-

FxaC_28g28700 gctgctttaatgtcttcaatttatgagaaacataaagatgaagatggttttctttatat-

FxaC_28g26540 ggagctgtaatgtctactatctatgatgacaagaaggacggtgatggatttctttacgt-

FxaC_26g27290 ggagctgtaatgtctaccatctatgatgacaagaaggacggtgatggatttctttacgt-

FxaC_26g27370 ggagctgtaatgtctaccatctatgatgacaagaaggacggtgatggatttctttacgt-

FxaC_25g27700 ggagctgtaatgtctactatctatgatgacaagaaggacggtgatggatttctttacgt-

FxaC_25g16410 ggagctgtaatgtctactatctatgatgacaagaaggacggtgatggatttctttacgt-

FxaC_27g20200 ggagctgtaatgtctactatctatgatgacaagaaggacggtgatggatttctttacgt-

FxaC_27g13830 ggagctgtaatgtctactatctatgatgacaagaaggacggtgatggatttctttacgt-

FxaC_17g00340 ggtgcaattatgtctgccatatatgaagagaagaaggatgaagatggctttctctacgt-

FxaC_18g46510 ggtgcaattatgtctgccatatatgaagagaagaaggatgaagatggctttctctacgt-

FxaC_19g00020 ggtgcaattatgtctgccatatatgaagagaagaaggatgaagatggctttctctatgt-

FxaC_17g00370 ggtgcaattatgtctgccatatatgaagagaagaaggatgaagatggctttctctacgt-

FxaC_25g13980 gctgctttaatgtcttcaatttatgagaaacataaggatgaagatggttttctttatat-

FvH4_4g21320 gcaagtcatatggattccatttacgaaacttacaaggaagatgatggatttctgtacat-

FvH4_1g26480 gctagtttgatgaactttgtctatgaatcgtacaaggacgaggatggatttctgtacat-

FxaC_1g32800 GCTAGTTTGATGAACTTTGTCTATGAATCGTACAAGGACGAGGATGGATTTCTGTACAT-

FxaC_2g36930 GCTAGTTTGATGAACTTTGTCTATGAATCGTACAAGGAGGAGGATGGATTTCTGTACTT-

FxaC_4g08940 GCTAGTTTGATGAGCTTTGTCTATGAATCGTACAAGGACGAGGATGGATTTCTGTACTT-

FxaC_13g24640 gcaagtcatatggattccatttacgaaacttacaaggaagatgatggatttctgtacat-

FxaC_14g17270 gcaagtcatatggattccatttttgaaacttacaaggaagatgatggatttctgtacat-

FxaC_15g17730 gcaagtcatatggattccatttatgaaacttacaaggaagatgatggatttctgtacat-

FxaC_27g31780 gctagtttgatgaactttgtctatgaatcgcacaaggacgaggatggatttctgtactt-

FvH4_6g19421 tctgctttgatgtcagatatttatgcagcaaatgctgatgaagatggatatctgtacatt

FxaC_21g44840 tctgctttgatgtcagatatttatgcagcaaatgctgatgaagatggatatctgtacatt

. *.* .* * .. . . ** *. ***** * .** **. *

FvH4_3g22920 ---gacatacagtggagaaaatacatttg--------------------gatctgtttg-

FvH4_3g01980 ---gacctacagtggagagaatgcctttg--------------------gatctcacta-

FvH4_6g43130 ---gacatacagtggcgagagcacatttg--------------------ggacgtgctg-

FvH4_7g09231 ---gacatacagtggcgagagcacgtttg--------------------ggatgtgctg-

FvH4_7g11130 ---ttcatacagtggtgaaaacacattcg--------------------gg------ta-

FvH4_5g00060 ---cacttacagcggtgagaacacattcg--------------------gttcccagat-

FxaC_12g01440 ---GACCTACAGTGGAGAGAATGTCTTTG--------------------GATCTCTTTA-

FxaC_9g01870 ---GACCTACAGTGGAGAGAATGTCTTTG--------------------GATCTCATTA-

FxaC_11g46890 ---GACCTACAGTGGAGAGAATGTCTTTG--------------------GATCTCATTA-

FxaC_12g25380 ---GACATACAGTGGAGAAAATACATTTG--------------------GATCCGTTTG-

FxaC_9g29910 ---GACATACAGTGGAGAAAATACATTTG--------------------GATCTATTAGT

FxaC_11g22890 ---gacatacagtggagaaaatacatttg--------------------gatccgtttg-

FxaC_12g25610 ---gacatacagtggagaaaatacatttg--------------------gatccgtttg-

FxaC_10g25330 ---gacatacagtggagaaaatacatttg--------------------gatcagtttg-

FxaC_12g48710 ---gacctacagtggagagaatgcctttg--------------------gatctcatta-

FxaC_10g04960 ---gacctacagtggagagaatgcctttg--------------------gatctcatta-

FxaC_23g56560 ---gacatacagtggcgagagtacgtttg--------------------ggacatgctg-

FxaC_22g12060 ---gacatacagtggcgagagcacgtttg--------------------gcacgtgctg-

FxaC_21g14090 ---gacatacagtggcgagagcacatttg--------------------ggac-------

FxaC_26g29480 ---gacatacagtggcgagagcacgtttg--------------------gaatgtgctg-

FxaC_28g28700 ---gacatacagtggcgagagcacgttag--------------------ggatgtgctg-

FxaC_28g26540 ---ttcatacagtggtgaaaacacattcg--------------------gg------ta-

FxaC_26g27290 ---ttcatacagtggtgaaaacacattcg--------------------gg------ta-

FxaC_26g27370 ---ttcatacagtggtgaaaacacatttg--------------------gg------ta-

FxaC_25g27700 ---ttcatacagtggtgaaaacacattcg--------------------gg------ta-

FxaC_25g16410 ---ttcatacagtggtgaaaacacattcg--------------------gg------ta-

FxaC_27g20200 ---ttcatacagtggtgaaaacacattcg--------------------gg------ta-

FxaC_27g13830 ---ttcatacagtggtgaaaacacattcg--------------------gg------ta-

FxaC_17g00340 ---cacttacagcggtgagaacacattcggttccc-----agatcccaagt------ta-

FxaC_18g46510 ---cacttacagcggtgagaacacattcggttccc-----agatcccaagt------ta-

FxaC_19g00020 ---cacttacagcggtgagaacacattcggttccc-----agatcccaagt------ta-

FxaC_17g00370 ---cacttacagcggtgagaacacattcggttccc-----agatcccaagt------ta-

FxaC_25g13980 ---gacatacaatggcgagagcacgtttggg-----------------atg------tg-

FvH4_4g21320 ---gtgttacagcagcgagaaaacctttg--------------------gc------ta-

FvH4_1g26480 ---gtgttacagtacggagaaaacctttggt-----------gatgcttgc------ta-

FxaC_1g32800 ---GTGTTACAGTACGGAGAAAACCTTTGGT-----------GATGCTTGC------TA-

FxaC_2g36930 ---GTGTTACAGTACGGAGAAAACCTTTGGT-----------GATGCTAGC------TA-

FxaC_4g08940 ---GAGTTACAGTACGGAGAAAACCTTTGGT-----------GATGCTAGC------TA-

FxaC_13g24640 ---gtgttacagcagcgagaaaacctttg--------------------gc------ta-

FxaC_14g17270 ---gtgttacagcagcgagaaaacctttg--------------------gc------ta-

FxaC_15g17730 ---gtgttacagcagcgagaaaacctttg--------------------gc------ta-

FxaC_27g31780 ---gtgttacagtacggagaaaacctttggt-----------gatgctagc------ta-

FvH4_6g19421 gccgatctacagtggagagggtgccttcgggtccctttaagaagcagcagc------ta-

FxaC_21g44840 gccgatctacagtggagagggtgccttcgggtccctttaagaagcagcagc------ta-

****... **... .. ** *

FvH4_3g22920 -----------------------a

FvH4_3g01980 -----------------------a

FvH4_6g43130 -----------------------a

FvH4_7g09231 -----------------------a

FvH4_7g11130 -----------------------g

FvH4_5g00060 -----------------------c

FxaC_12g01440 -----------------------A

FxaC_9g01870 -----------------------A

FxaC_11g46890 -----------------------A

FxaC_12g25380 -----------------------A

FxaC_9g29910 AATGTGATACGATATATTATATAA

FxaC_11g22890 -----------------------a

FxaC_12g25610 -----------------------a

FxaC_10g25330 -----------------------a

FxaC_12g48710 -----------------------a

FxaC_10g04960 -----------------------a

FxaC_23g56560 -----------------------a

FxaC_22g12060 -----------------------a

FxaC_21g14090 ------------------------

FxaC_26g29480 -----------------------a

FxaC_28g28700 -----------------------a

FxaC_28g26540 -----------------------g

FxaC_26g27290 -----------------------g

FxaC_26g27370 -----------------------g

FxaC_25g27700 -----------------------g

FxaC_25g16410 -----------------------g

FxaC_27g20200 -----------------------g

FxaC_27g13830 -----------------------g

FxaC_17g00340 -----------------------g

FxaC_18g46510 -----------------------g

FxaC_19g00020 -----------------------g

FxaC_17g00370 -----------------------g

FxaC_25g13980 -----------------------c

FvH4_4g21320 -----------------------a

FvH4_1g26480 -----------------------a

FxaC_1g32800 -----------------------A

FxaC_2g36930 -----------------------A

FxaC_4g08940 -----------------------A

FxaC_13g24640 -----------------------a

FxaC_14g17270 -----------------------a

FxaC_15g17730 -----------------------a

FxaC_27g31780 -----------------------a

FvH4_6g19421 -----------------------g

FxaC_21g44840 -----------------------g
