# Supplementary material for: Autophagy Is Required for Strawberry Fruit Ripening
Source: Front Plant Sci. 2021 Aug 27;12:688481. doi: 10.3389/fpls.2021.688481 (PMC8429490; doi:10.3389/fpls.2021.688481)
Supplement: Supplementary Table 1 — (S1) List of primers used in this work. (S2) Autophagy related genes found in F. vesca genome. [file Table_1.DOCX]

**Supplemental Table S1.** List of primers used in this work.

| **RNAi** |  |
| --- | --- |
| *FaATG5*_Attb1_F | 5´-GGGGACAAGTTTGTACAAAAAAGCAGGCTTCTGTGCACAACCTGAGAGA-3´ |
| *FaATG5*_Attb2_R | 5´-GGGGACCACTTTGTACAAGAAAGCTGGGTAATATACTCGGTCGGGATCTTGG-3´ |
| *FaATG7*_Attb1_F | 5´-GGGGACAAGTTTGTACAAAAAAGCAGGCTCTGTTGCTTGATCCTCCAGC-3´ |
| *FaATG7*_Attb2_R | 5´-GGGGACCACTTTGTACAAGAAAGCTGGGTATGCAAAACCACGAGTCTCTC-3´ |
| **qRT-PCR** |  |
| *FaNBR1*_F | 5´-GAGGATCGATGTGCAAAGGA-3´ |
| *FaNBR1*_R | 5´-GAGTAGTTCACGGAGAGGCA-3´ |
| *FaVPS34*_F | 5´-CAACTGGTTCAAGCACTTCGA-3´ |
| *FaVPS34*_R | 5´-GCGAAGAAAGCTAGCCAACT-3´ |
| *FvATG5*_F | 5´-AACCGGCCTGTTGAGATACA-3´ |
| *FvATG5*_R | 5´-TTTGCTCTTCCTCACCACCT-3´ |
| *FvATG7*_F | 5´-GAGACTCGTGGTTTTGCAGA-3´ |
| *FvATG7*_R | 5´-ACCCCACTGCATTAGGTGTA-3´ |

**Supplemental Table S2**. Autophagy related genes found in *F. vesca* genome.

| **Gene** | **Orthologs in *A. Thaliana*** | **Orthologs in *F. vesca*** | ***Arabidopsis***  **AGI code** | ***F. vesca***  **gene name** |
| --- | --- | --- | --- | --- |
| *ATG1* | 3 | 3 | AT3G61960  AT3G53930  AT2G37840 | FvH4_7g17860 FvH4_7g17850 FvH4_6g12940 |
| *ATG2* | 1 | 1 | AT3G19190 | FvH4_6g11120 |
| *ATG3* | 1 | 1 | AT5G61500 | FvH4_7g09530 |
| *ATG4* | 2 | 2 | AT3G59950  AT2G44140 | FvH4_2g06800 FvH4_2g06790 |
| *ATG5* | 1 | 1 | AT5G17290 | FvH4_5g28670 |
| *ATG6* | 1 | 1 | AT3G61710 | FvH4_7g16330 |
| *ATG7* | 1 | 1 | AT5G45900 | FvH4_3g07910 |
| *ATG8* | 9 | 9 | AT3G15580  AT2G45170  AT2G05630  AT4G16520  AT4G21980  AT3G60640  AT4G04620  AT1G62040  AT3G06420 | FvH4_7g11130 FvH4_5g00060 FvH4_3g01980 FvH4_3g22920 FvH4_1g26480 FvH4_4g21320 FvH4_6g43130 FvH4_6g19421 FvH4_7g09231 |
| *ATG9* | 1 | 1 | AT2G31260 | FvH4_2g21710 |
| *ATG10* | 1 | 1 | AT3G07525 | FvH4_2g17490 |
| *ATG11* | 1 | 1 | AT4G30790 | FvH4_4g10150 |
| *ATG12* | 2 | 1 | AT1G54210  AT3G13970 | FvH4_6g34660 |
| *ATG13* | 2 | 2 | AT3G49590  AT3G18770 | FvH4_7g33350 FvH4_1g23440 |
| *ATG18* | 8 | 5 | AT1G03380  AT1G54710 AT2G40810 AT3G56440 AT3G62770 AT4G30510 AT5G05150 AT5G54730 | FvH4_2g25170 FvH4_6g06370 FvH4_6g06350 FvH4_7g26300 FvH4_6g06351 |
| *NBR1* | 1 | 1 | AT4G24690 | FvH4_5g06270 |
| *VPS34* | 1 | 1 | AT1G60490 | FvH4_4g34140 |
